# Silk Fibroin-Based Materials for Catalyst Immobilization

**DOI:** 10.3390/molecules25214929

**Published:** 2020-10-24

**Authors:** Shanshan Lv

**Affiliations:** State Key Laboratory of Organic-Inorganic Composite Materials, College of Chemical Engineering, Beijing University of Chemical Technology, 15 BeisanhuanDong Road, Chaoyang District, Beijing 100029, China; lvshanshan@mail.buct.edu.cn

**Keywords:** silk fibroin, enzyme immobilization, metal, metal oxide, catalyst

## Abstract

Silk fibroin is a widely and commercially available natural protein derived from silkworm cocoons. Thanks to its unique amino acid composition and structure, which lead to localized nanoscale pockets with limited but sufficient hydration for protein interaction and stabilization, silk fibroin has been studied in the field of enzyme immobilization. Results of these studies have demonstrated that silk fibroin offers an important platform for covalent and noncovalent immobilization of enzymes through serving as a stabilization matrix/support with high retention of the biological activity of the enzymes of interest. In the hope of providing suggestions for potential future research directions, this review has been written to briefly introduce and summarize key advances in silk fibroin-based materials for immobilization of both enzymes/biocatalysts (including alkaline phosphatase, β-glucosidase, glucose oxidase, lipase, urease, uricase, horseradish peroxidase, catalase, xanthine oxidase, tyrosinase, acetylcholinesterase, neutral protease, α-chymotrypsin, amylase, organophosphorus hydrolase, β-galactosidase, carbonic anhydrase, laccase, zymolyase, phenylalanine ammonia-lyase, thymidine kinase, and several others) and non-enzymatic catalysts (such as Au, Pd, Fe, α-Fe_2_O_3_, Fe_3_O_4_, TiO_2_, Pt, ZnO, CuO, Cu_2_O, Mn_3_O_4_, and MnO_2_).

## 1. Introduction

**Figure 1:**
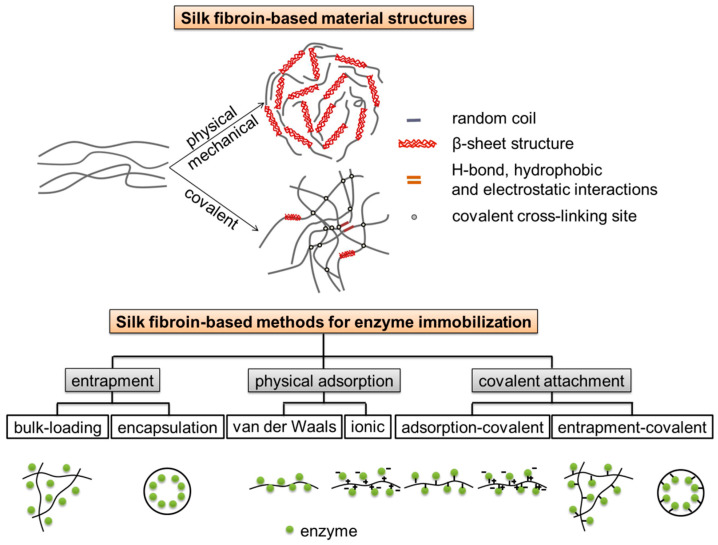
Silk fibroin-based materials for catalyst immobilization. Schematic illustration of silk fibroin-based material structures and silk fibroin-based methods for enzyme immobilization [13].

Silk fibroin, which gives silk unique physiochemical and mechanical properties, is naturally derived from domesticated *Bombyx mori* silkworm cocoon silk [1]. Silk fibroin is relatively cheap and readily available, and has been utilized for traditional textile applications, surgical sutures, and beyond [2,3,4]. Silk fibroin is a macromolecular protein containing large amounts of glycine, alanine, and serine [5,6], as well as readily activated chemical groups, such as tyrosyl/phenol, sulfhydryl, and imidazole groups [7,8]. Because of its unique amino acid sequence, silk fibroin displays conformational transition from water soluble silk I structure to water-insoluble silk II structure in response to environmental stimuli (Figure 1). Silk fibroin can be easily developed into various forms under mild, ambient, aqueous conditions, such as fibers, powders (microspheres/nanoparticles), films, membranes, gels, hydrogels, and scaffolds. In particular, silk fibroin possesses many important attractive inherent features which suggest utility as an enzyme stabilization matrix [9], such as relative inexpensiveness, robust mechanical properties, excellent biocompatibility, high microbial resistance, controllable biodegradability, suitable stability due to extensive network of hydrogen bond cross-links [10,11], and unique block copolymer structure consisting of large organized hydrophobic domains (i.e., self-assembled crystalline β-sheets) and small flexible hydrophilic spacers [12], which could provide a stabilizing microenvironment for enzyme stabilization [13]. Several different forms of silk fibroin have been investigated to serve as supports for enzyme immobilization. Immobilization of bioactive enzymes could be built into diagnostic and therapeutic applications; immobilization of industrial enzymes would allow reuse of expensive enzymes for expanded utility in industrial processes like food and cosmetics [13].

In 1976, Inada et al. applied for a patent on enzyme absorption by regenerated silk fibroin fibers made from liquid silk in vivo [14]. Since then, there have been numerous papers on enzyme-immobilized silk fibroin, which can be divided into three main approaches, including physical adsorption (by van der Waals and/or hydrophobic interactions), entrapment (via bulk-loading and encapsulation in microspheres/nanoparticles), and covalent attachment, respectively (Figure 1). The choice or combination of immobilization approaches depends on application scenarios/purposes and properties of the enzymes of interest [13]. Enzymes immobilized in silk fibroin have been characterized by means of spectrophotometry (such as Fourier transform infrared spectroscopy (FT-IR), nuclear magnetic resonance (NMR), electron spin resonance (ESR)) and microscopy (such as scanning electron microscopy (SEM) and atomic force microscopy (AFM)), and evaluated in biosensors showing high stability. A review of use of silk fibroin as supports for enzyme immobilization was published [15], and a review of physical and chemical aspects of stabilization of small molecules and proteins in silk fibroin biomaterials, covering horseradish peroxidase, glucose oxidase, and lipase as well as several other enzymes, was also published before [13]. However, in the past five years, there were few reviews of recent advances on the immobilization of enzymes on silk fibroin; even less reviews of immobilization of non-enzymatic catalysts using silk fibroin as a support have been reported.

This review covers silk fibroin-based immobilization and stabilization of both enzymes/biocatalysts and non-enzymatic catalysts during preparation, operation, and long-term storage. It is of note that many readers may have encountered some contents of the immobilization of enzymes in previous studies; we hope to offer a relatively comprehensive list for readers interesting in the silk fibroin-based immobilization approach to easily find which enzymes have been investigated, as well as the corresponding immobilization methods and explored applications. Previously reported silk fibroin-stabilized enzymes in diverse forms (including fibers, films, scaffolds/sponges, gels, and powders) were arranged roughly in chronological order to show the development of silk fibroin for enzyme immobilization (Table 1, Section 2). A variety of silk fibroin-supported metal and metal oxide catalysts were arranged according to the metal elements in the catalysts, demonstrating capability of silk fibroin in maintaining activities of non-enzymatic catalysts (Table 2, Section 3). Challenges in the silk fibroin-based immobilization approach were also discussed, and possible directions for future research and development were suggested.

## 2. Immobilization of Enzymes/Biocatalysts

### 2.1. Alkaline Phosphatase

Grasset et al. reported using woven silk as a carrier for immobilization of alkaline phosphatase and aspartate aminotransferase in 1977 [7]. The enzymes were fixed by physical absorption and covalent bond through acid methylation [7], glutaraldehyde [8], and the azide/diazo-coupling technique [8,16]. These immobilization methods were easy and allowed maintain of enzyme activity [7]. Cordier et al. investigated the immobilization of ribonuclease A [16], glycyl-tRNA-synthetase, and industrial rennet onto woven silk using the diazo-coupling method. The immobilized ribonuclease A retained 63% activity after 7.2 months of storage in 0.1 M NaCl at 0–48 °C [16].

Asakura et al. reported immobilization of alkaline phosphatase on silk fibroin fiber by covalent bond through the diazo and cyanogen bromide coupling methods, characterized the immobilized enzyme (Michaelis constant K_m_ and maximum activity V_m_), and optimized the immobilization conditions (such as pH, enzyme concentration, reaction time and temperature). They found that immobilization shifted the optimum pH of the enzyme to the acid side, improved the thermal stability of the enzyme above 50 °C, and maintained activity over a long period [17]. Demura et al. investigated the immobilized enzyme and silk fibroin during the reaction process and after the reaction by scanning electron microscopy (SEM), electron spin resonance (ESR), and nuclear magnetic resonance (NMR). In addition, the activity of the immobilized alkaline phosphatase could be much improved by pretreatment of silk fibroin fibers by low-temperature plasmas [18].

**Figure 2:**
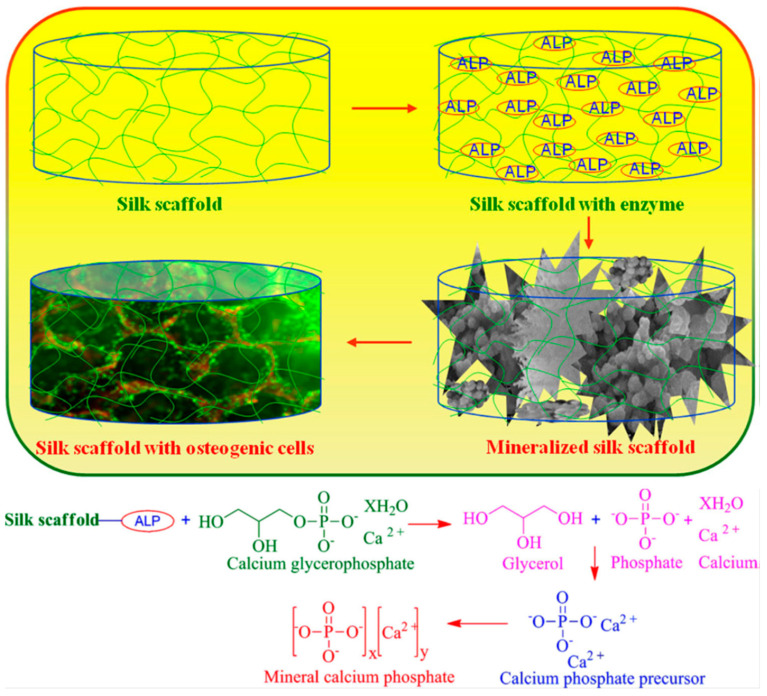
Proposed reaction mechanism of calcium phosphate with alkaline phosphatase (ALP) soaked into silk fibroin scaffolds. Reprinted with permission from REFERENCE [19]. Copyright (2014) John Wiley and Sons.

Samal, Dubruel, and Kaplan studied alkaline phosphatase mediated homogeneously formation of apatite minerals on porous silk fibroin scaffolds under physiological conditions (Figure 2). It was explained that the active metallic sites of alkaline phosphatase interacted with the acid (-COOH) and amino (-NH_2_) groups of silk fibroin, as a result, alkaline phosphatase was entrapped within the porous silk fibroin scaffold. The immobilized alkaline phosphatase further induced deposition of calcium phosphate/mineralization. It was found that the mineral structure varied at different alkaline phosphatase concentrations, and that 20 mg·mL^−1^ alkaline phosphatase mineralized silk fibroin scaffolds maximized MC3T3 osteoblast cell differentiation to obtain bone-like tissue in vitro. These results demonstrated a simple and efficient strategy to fabricate mineralized scaffolds for bone tissue engineering applications [19].

### 2.2. β-Glucosidase

Fukui and co-workers reported immobilization of β-glucosidase in insoluble silk fibroin membrane by drying silk fibroin and enzyme mixture and subsequent ethanol treatment in 1978. The immobilized β-glucosidase retained 47% activity using p-nitrophenyl-β-d-glucopyranoside as a substrate, and showed improved stability against heating, electrodialysis, protease/papain treatment, and storage. It was found that immobilization increased the activation energy of the enzyme slightly, but did not significantly change pH dependency of the enzyme [20].

Puri and co-workers studied the immobilization of β-glucosidase by adsorption to eri silk fibroin microparticles with a 62% immobilization yield. The ultrafine powders were produced by a wet milling and spray drying process without using chemicals. The results showed that immobilization changed the optimum pH (from 4.0 to 5.0 at 60 °C) and kinetics (the Michaelis constant K_m_ from 0.16 to 0.27 mM) of the enzyme. The immobilized enzyme exhibited enhanced stability under thermal denaturation at 70 °C, and good reusability maintaining more than 50% of initial activity for up to eight cycles. Porous crystalline silk fibroin microparticles provide a promising support for immobilization of β-glucosidase for effective cellobiose hydrolysis for potential application in biofuel production [21].

Zhang and co-workers studied covalent immobilization of naringinase, which is a bienzyme of 𝛼-l-rhamnosidase and flavonoid-𝛽-glucosidase, on silk fibroin nanoparticles using glutaraldehyde. Silk fibroin nanoparticles were produced by rapidly adding an aqueous solution of regenerated silk fibroin into excess organic solvents (acetone). The activities in naringin hydrolysis of the immobilized enzyme were analyzed by high-performance liquid chromatography (HPLC), showing similar kinetics and optimum reactive temperature to those of the free enzyme. In addition, the immobilized enzyme could be easily separated and recovered by simple centrifugation, and be used repeatedly; after 8 repeated reaction cycles (8–10 h at 55 °C for each cycle), the immobilized enzyme retained about 70% residual activity. These results demonstrated a highly efficient processing technology to produce low-cost immobilized naringinase, showing great potential in industrial naringin-containing juice debittering processing [22].

### 2.3. Glucose Oxidase

Kuzuhara et al. reported immobilization of glucose oxidase in a silk fibroin membrane, which were treated with 80% methanol (and glutaraldehyde to serve as a control). The immobilized glucose oxidase recovered 98% activity with 3.71 × 10^−3^ U glucose oxidase immobilized in 1.1 mg silk fibroin membrane, maintain activity with only 0.05% enzyme leakage over one month, and exhibited improved stability to pH and heat at 40–60 °C, retaining 100% and 97% activity after 20 min at 60 °C and 70 °C, respectively, while free enzyme lost activity at above 60 °C. Infrared spectrum of the immobilized enzyme indicated that structural configuration was random coil inside the silk fibroin membrane and anti-parallel β-sheet on the surface [23].

Demura at al. studied immobilization of glucose oxidase in regenerated silk fibroin membrane by simple physical treatment, such as stretching (i.e., uniaxially drawing by placing on a stretcher), compressing and standing under high humidity (i.e., hydration by placing in a desiccator of 96% relative humidity for 17 h) and methanol-immersion [24,25,26]. The results showed that the configuration transition of silk fibroin (from random coil to anti-parallel β-sheet) led to a similar simultaneous insolubilization of the membrane, and thereby immobilization of the enzyme, regardless of methanol-immersion and the physical treatment. However, the activity and stability of the immobilized enzyme depended on the characteristics of the silk fibroin membrane, for instance, the fraction of anti-parallel β-sheet and permeability of glucose and oxygen. Immobilization improved stability of the enzymes against pH and heat, although the optimum pH shifted to around pH 7.0. Furthermore, the glucose oxidase-immobilized silk fibroin membrane was applied in the development of the glucose sensor in a steady state analysis system based on an oxygen electrode, obtaining a linear relationship between output and glucose concentration within 0–5 mM for 0.2% enzyme in membrane, 0–9 mM for 0.02% enzyme in membrane, and 0–25 mM for 0.002% enzyme in membrane [25,26].

**Figure 3:**
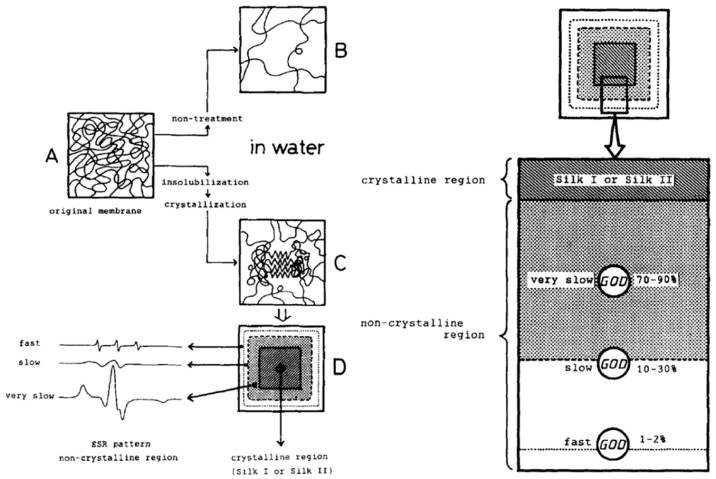
Schematic representation of immobilization of glucose oxidase in silk fibroin membrane. Left: water-insoluble membrane (**A**) in dry state, (**B**) in aqueous solution, (**C**) swollen by water, and (**D**) relation between ESR spectra patterns and the non-crystalline region in the spin-labeled silk fibroin membrane. Right: proposed model of glucose oxidase in the membrane. Reprinted with permission from REFERENCE [32]. Copyright (2004) John Wiley and Sons.

Moreover, Asakura et al. reported glucose oxidase was immobilized on nonwoven fabrics of silk fibroin and silk fibroin gel, when applied in the glucose sensor, four times increase in sensitivity was observed, compared with glucose oxidase immobilized in the silk fibroin membrane [27]. Asakura and co-workers tried to clarify the enzyme reaction mechanism in the silk fibroin membrane/gel using nuclear magnetic resonance (NMR), particularly high-resolution multi-nuclei NMR and ^13^C cross-polarization/magic angle spinning (CP/MAS) NMR, and electron spin resonance (ESR, Figure 3) to characterize the state of substrate and immobilized enzyme [28,29,30,31,32,33,34,35,36,37]. For example, diffusion coefficient of small paramagnetic substrate molecules into silk fibroin gel was determined [38]. In addition, Asakura and co-workers reported immobilization of invertase in silk fibroin powders, which were prepared from aqueous solutions of silk fibroin by different insolubilization methods. Immobilization improved thermal stability of invertase. In addition, the structures of silk fibroin powders (spin-labeled at the OH group of the tyrosine side chain) in the swollen state in water were characterized by electron spin resonance (ESR) and ^13^C nuclear magnetic resonance (NMR), and the dry state by Fourier transform infrared spectroscopy (FT-IR). The results indicated that insolubilization affected the conformational transition of silk fibroin from the random coil to antiparallel β-sheets [33].

Yu and co-workers reported immobilization of glucose oxidase in regenerated silk fibroin from waste silk from a silk mill. The glucose oxidase-immobilized membrane was analyzed by Fourier transform infrared spectroscopy (FT-IR), scanning electron microscopy (SEM), and electronic absorption bands. The FT-IR spectra showed a composite of the absorption bands of structural characteristics of both silk fibroin and glucose oxidase. The electronic absorption bands and SEM showed that glucose oxidase aggregates in the membrane. Then the glucose oxidase-immobilized membrane was applied in developing tetrathiafulvalene-mediated [39] and ferrocene-mediated [40] glucose sensors, based on transferring electrons between the immobilized enzyme and a glassy carbon electrode. The immobilization protected glucose oxidase against auto-inactivation and thermo-deactivation, resulting in a fast response time and good stability of the sensors. Furthermore, in order to improve the mechanical property of the membrane, they reported blend membranes of regenerated silk fibroin and poly (vinyl alcohol) PVA at a ratio of 1:5 for the immobilization of glucose oxidase with ethanol treatment. The glucose oxidase-immobilized membrane were applied in construction of a glucose sensor with a hydrogen peroxide probe, which determined glucose with a fast response time, good reproducibility, high storage and operational stability. In addition, poly (ethylene glycol) PEG was added as a reagent for making holes in the membrane to decrease resistance to material transport. The glucose oxidase-immobilized membrane was applied in construction of a glucose sensor through coupling the Clark oxygen electrode. The resultant glucose sensor showed improved stability in a broad range of pH and temperature [41,42,43].

Zhang and co-workers reported immobilization of glucose oxidase in methanol-treated silk fibroin films with high storage stability; activity maintained in broad pH (5.0–10.0) and temperature (20 to 50 °C) ranges, over two years (when stored at 4 °C). Based on the glucose oxidase-immobilized silk fibroin membrane, oxygen electrode and a temperature control system, a glucose sensor was developed, showing a broad range of linear response for glucose, and capable of detecting over 60 biosamples per h, and over 1000 repeated times for biosamples [44].

Kaplan and co-workers reported untreated silk fibroin films loaded with 1 wt% glucose oxidase, showing good long-term stability over months when stored at 4 °C and 25 °C. Moreover, it was found that glucose oxidase showed higher stability in untreated films compared with methanol-treated films, probably due to stacked β-sheet and thereby more hydrophobic and restrictive structures of methanol-treated films [9,45].

**Figure 4:**
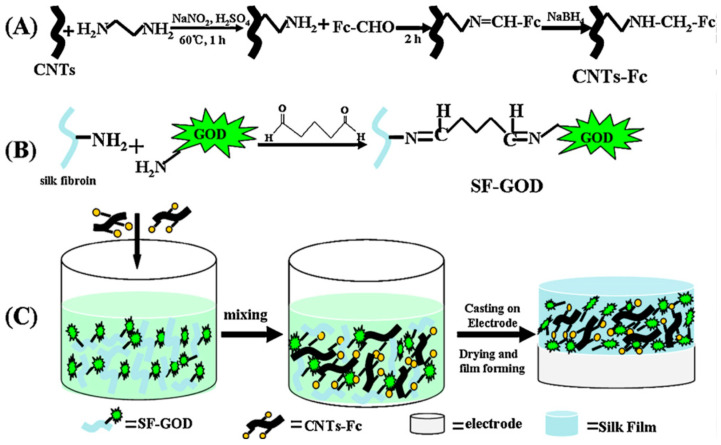
Schematic illustration of preparation of (**A**) ferrocenecarboxaldehyde-immobilized ethylenediamine-functionalized carbon nanotubes (CNTs-Fc), (**B**) glucose oxidase-immobilized silk fibroin (SF-GOD), and (**C**) CNTs-Fc/SF-GOD-modified electrode. Reprinted with permission from REFERENCE [46]. Copyright (2012) Elsevier.

Chen and co-workers reported development of a high-performance bioanode based on the composite of ferrocenecarboxaldehyde-immobilized ethylenediamine-functionalized carbon nanotubes and glucose oxidase-immobilized silk fibroin films (Figure 4). The resultant electrode exhibited good activity and excellent stability, and used as the bioanode for glucose/O_2_ biofuel cell [46].

**Figure 5:**
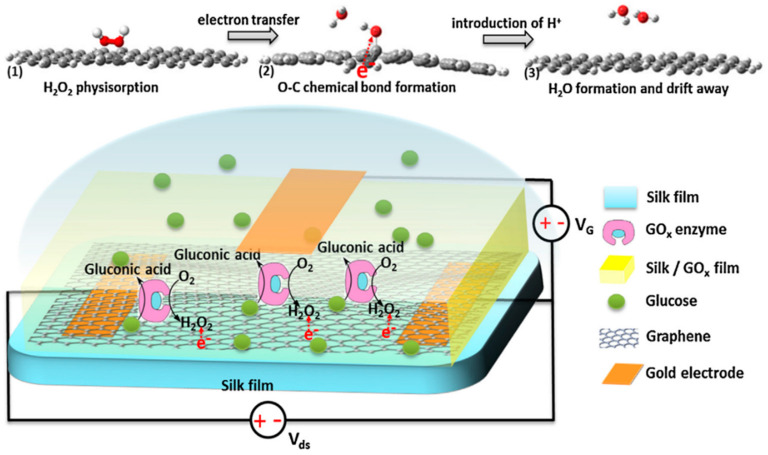
Schematic illustrations of glucose oxidase-immobilized silk fibroin film functionalized graphene field effect transistor and proposed mechanism of glucose sensing process. Reprinted with permission from REFERENCE [47]. Copyright (2014) Elsevier.

Pak and co-workers reported a glucose oxidase-immobilized silk fibroin film on graphene field effect transistor (FET) as a glucose sensor (Figure 5). The sensor exhibited excellent selectivity and sensitivity, a linear detection range of 0.1 to 10 mM glucose concentration, which was useful for diabetes diagnostics. This study provided a promising long-term stable, flexible, portable, wearable, biocompatible and implantable silk fibroin-based sensor for patch type, highly selective, sensitive, and continuous real-time glucose level monitoring applications [47].

Zheng and Lu illustrated design and performance of a glucose oxidase-immobilized silk/D-sorbitol pyramidal microneedle integrated with platinum (Pt) and silver (Ag) wire-based minimally invasive electrochemical biosensor for continuous glucose monitoring. The immobilized glucose oxidase displayed high stability, quick response at low glucose concentrations, and a linear correlation within 1.7–10.4 mM glucose concentration [48].

### 2.4. Lipase

Asakura and co-workers reported immobilization of lipase in silk fibroin membrane. By methanol immersion treatment, no leakage of the immobilized enzyme was observed. After being immobilized, the optimum temperature shifted to 50 °C from 20 °C of the free enzyme, and stability improved at 50 °C with less than 2.5% inactivation of that of the free enzyme; the optimum pH also shifted to higher values on the alkaline side as compared to that of the free enzyme. The immobilized enzyme and tributyrin as the substrate was further studied by ^13^C NMR, implying that the stabilization effect was mainly attributed to the unique structure of silk II crystalline [49].

Kaplan and co-workers reported immobilization of glucose oxidase, horseradish peroxidase and lipase in silk fibroin films with respect to long-term storage, maintaining significant activity (>40% and up to 100%) over 10 months storage at temperatures ranging from 4 to 37 °C. The effect of silk film processing methods on the enzyme stability was investigated and optimized, showing that the immobilized enzymes were more stable in silk films without methanol treatment than those with methanol treatment. Silk fibroin structural changes, enzyme distribution and denaturation/renaturation were investigated, suggesting that the stabilization effect was correlated to intermolecular interaction between silk fibroin and enzymes, as well as enzyme sensitivity to oxidation and hydrophobic-hydrophilic interfaces in the microenvironment established by the unique structure of silk fibroin [9,45].

Tan and co-workers studied immobilization of lipase by silk fibers in the form of woven fabric. The hydrophilic/hydrophobic properties of the silk fibers were tuned by functionalization with methyl groups via treatment with amino-functional polydimethylsiloxane (PDMS). The effects of pH, temperature, and organic media with different water content range from 1 to 10% (*v*/*v*) on the activity and stability of lipases were investigated and compared. It was found that lipase immobilized on hydrophobic silk fibers showed better activity and significantly improved operational stability (over a wider pH range and a shift in optimum temperature) in olive oil hydrolysis and dodecanoic acid esterification, compared with those on hydrophilic silk fibers and the free lipase. In addition, even after 27 repeated cycles, the yield maintained around 97%. These results indicated that woven silk fabrics could be a potential lipase immobilization support for industrial applications [50].

Porto and co-workers investigated immobilization of lipase in a blend of gelled silk fibroin-calcium alginate spheres for enzymatic kinetic resolution of chlorohydrins as substrates. The enantioselectivity was sufficient for simple and low cost synthesis of alcohols and acetates in good yields and high enantiomeric purities [51]. Then, the immobilized lipase was applied to catalyze transesterification of soybean oil with ethanol under different conditions for industrial production of biodiesel (fatty acid ethyl esters). The optimized conditions were found to be 150 mg of soybean oil, 450 μL of ethanol, and 30 wt% immobilized lipase, when reacted at 32 °C for 48 h, resulting in a biodiesel yield of 42%, and the immobilized lipase maintained reusable in four repeated cycles [52]. Furthermore, lipase immobilized on silk fibroin spheres was used in enzymatic kinetic resolution of halohydrins, to obtain optically active chiral epoxides (in enantiomerically pure form). The immobilized lipase exhibited good activities, high selectivity and high enantiomeric excess up to 99%, reinforcing versatility of the biodegradable silk fibroin as an eco-friendly and efficient support for heterogeneous catalysts [53].

Goswami and co-workers reported immobilization of lipase on silk fibroin fibers via covalent cross-linking with glutaraldehyde for hydrolysis of sunflower oil for production of fatty acids. A maximum lipase loading of 59 U·g^−1^ silk fiber was obtained. The immobilized lipase showed improved stability up to 2 month at 4 °C, and could be reused in 4 repeated cycles [54]. Moreover, cholesterol oxidase was covalently immobilized onto silk fibroin fiber in the form of porous woven mats. Using *N*-ethyl-*N*’-(3-dimethylaminopropyl) carbodiimide and *N*-hydroxysuccinimide ligand chemistry, the loading efficiency was 70%, and the maximum loading was 0.046 U·cm^−2^ woven mats. The immobilized enzyme displayed remarkably storage stability up to 13 months at 4 °C, and could be reused in 25 repeated cycles (for a period of 6 h). These results demonstrated that silk fibroin provided a suitable enzyme immobilizing matrix with good stability, selectivity, sensitivity, and reproducibility promising for future development of biosensors [55].

### 2.5. Urease

Deng and co-workers reported immobilization of urease in silk fibroin (obtained directly from the middle division of the silk gland in full-grown larvae of living *Bombyx mori*) membrane by methanol immersion based on structural transition from random coil to anti-parallel β-sheet for construction of an urea electrode. The resultant urease-immobilized silk fibroin electrode exhibited high activity, short response time, superior thermal stability, excellent reproducibility (from batch-to-batch), and favorable sufficient lifetime (at room temperature for 3 months in air) [56].

**Figure 6:**
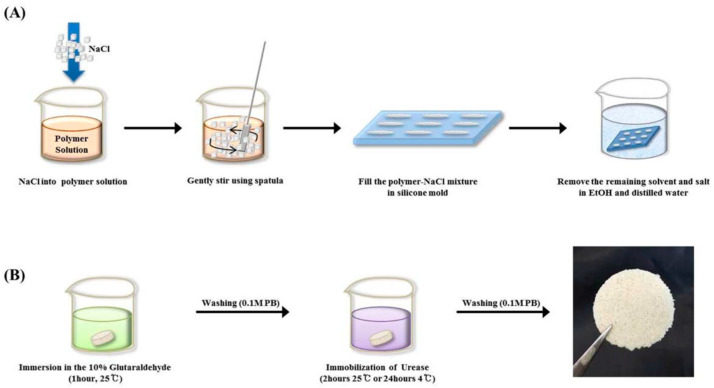
Schematic presentation of fabrication of porous silk fibroin filters by (**A**) the salt leaching method and (**B**) subsequent immobilization of urease. Reprinted with permission from REFERENCE [57]. Copyright (2016) John Wiley and Sons.

Park and co-workers studied immobilization of urease in silk fibroin membrane and evaluated its performances in terms of urea removal efficacy as a filtering system for peritoneal dialysate regeneration in wearable artificial kidney (Figure 6). The urease-immobilized silk fibroin membranes showed high porosity (as revealed by SEM-EDX analysis and porosity test) and water-binding abilities, when employed as filters, could remove 60% of urea in 50 mg·dL^−1^ urea solution, and 90% of urea in the peritoneal dialysate after 24 h filtration. The results suggested that silk fibroin membranes provide a suitable condition for efficient enzyme immobilization and urea removal, promising in peritoneal dialysate regenerative systems [57].

### 2.6. Uricase

Zhang et al. reported immobilization of uricase in silk fibroin membrane, which together with and an oxygen electrode was implanted in an amperometric urate sensor in a flow injection system for clinical diagnoses and fermentation. The urate sensor with a size of less than 10 mm in diameter could be stored for over 2 years, stable in phosphate buffer (from pH 7.80 to pH 9.00) for 3–4 months, used repeatedly for more than 1000 times for measurement of biosamples such as human serum or urine, and measure more than 60 biosamples per hour for rapidly determining uric acid level [58].

### 2.7. Horseradish Peroxidase

Asakura and co-workers reported immobilization of peroxidase in silk fibroin (obtained directly from silk larvae) membrane without any chemical cross-linking agents, and applied to biophotosensors for determining the concentration of hydrogen peroxide generated by the luminol reaction. It was found that the photocurrent from the photodiode increased linearly with the concentration of hydrogen peroxide in aqueous solution. In addition, the response time depended on the characteristics of the peroxidase-immobilized silk fibroin membrane; the response was fast and the intensity of the photocurrent was strong when peroxidase was distributed asymmetrically in the membrane, compared with peroxidase uniformly distributed [59].

Yu and co-workers investigated regenerated silk fibroin membrane prepared from waste silk for immobilization of horseradish peroxidase. The horseradish peroxidase-immobilized silk fibroin membrane and a glassy carbon electrode were used to fabricate an amperometric H_2_O_2_ sensor using phenazine methosulfate as the electron transfer mediator. The sensor displayed high sensitivity with a detection limit of 1.0 × 10^−7^ M, and a response time of less than 5 s for optimum analytical performance [60]. It was found that other organic agents such as meldola blue, methylene blue (with a response time of less than 40 s and storage stability over 2 months at 4 °C) [61,62], tetrathiafulvalene (with good reproducibility) [63], cresyl fast violet, catechol violet, methylene violet, brilliant cresyl blue, toluidine blue, and methylene green [64] could also provide suitable effective electron shuttles in development of a reliable, low-cost, highly sensitive sensor for H_2_O_2_ promising for practical application in bioanalysis. In addition, a H_2_O_2_ biosensor with remarkable long-term stability in organic media for organic-phase enzymatic assay was also prepared based on ferrocyanide-mediated electron transfer between a glassy carbon electrode with immobilized horseradish peroxidase and other enzymes such as dehydrogenases, glucose oxidase and cholesterol oxidase in water-isopropyl alcohol system [65].

Moreover, in order to improve the mechanical properties, a blend membrane of regenerated silk fibroin and poly (vinyl alcohol) PVA was prepared for the immobilization of peroxidase by ethanol treatment for fabrication of a ferrocene-mediated H_2_O_2_ sensor [66]. This method could be extended to other mediators such as meldola blue (with a detection of limit of 0.1 pM) [67], methylene blue (with a detection limit of 5.0 pM, operating and storage stabilities as well as rapid response time) [68], phenazine methosulfate, cresyl fast violet, catechol violet, methylene green [69], brilliant cresyl blue, toluidine blue, and 3-pnaphthoyl-Nile Blue A. Furthermore, immobilization of a bi-enzyme system of lactate oxidase and horseradish peroxidase in a blend membrane of regenerated silk fibroin and poly (vinyl alcohol) (PVA) was studied for construction of an amperometric phenazine methosulfate-mediated sensor highly selective and sensitive to lactate with a rapid response time within 20 s [70].

Oliva et al. investigated intermediates in the enzymatic oxidation reaction of silk fibroin with H_2_O_2_ in the presence of horseradish peroxidase by electron paramagnetic resonance (EPR) and ultraviolet/visible (UV/Vis) spectrophotometry, demonstrating that in aqueous solutions tyrosine side chains of silk fibroin as electron donor reacted with horseradish peroxidase and H_2_O_2_ generating phenoxyl radicals [136].

**Figure 7:**
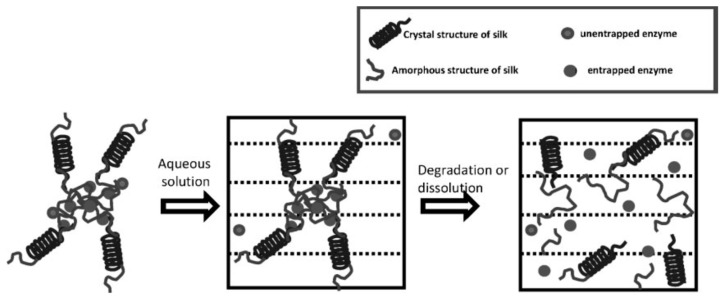
Schematic illustration of proposed model of enzyme-loaded insoluble silk fibroin films. Reprinted with permission from REFERENCE [72]. Copyright (2010) John Wiley and Sons.

**Figure 8:**
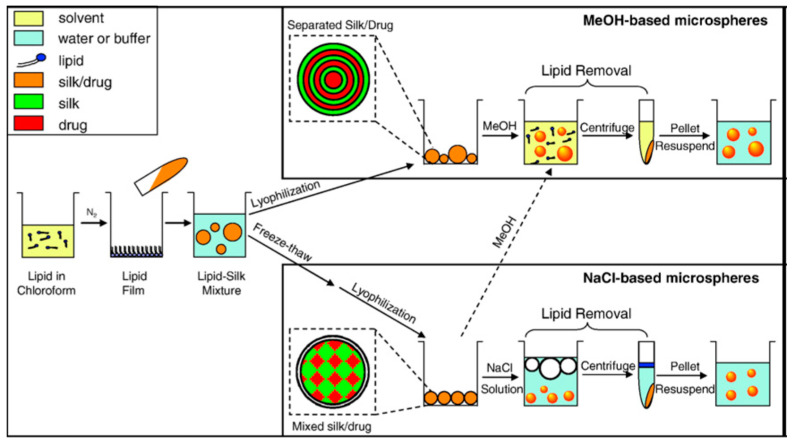
Schematic illustration of preparation process of silk fibroin microspheres. Reprinted with permission from REFERENCE [74]. Copyright (2007) Elsevier.

Kaplan and co-workers reported covalent immobilization of horseradish peroxidase in gradient manner (with bilaterally symmetrical patterns) in three-dimensional (3D) silk fibroin scaffolds/sponges using water-soluble carbodiimide chemistry [71]. Kaplan and co-workers also reported increasing horseradish peroxidase activity and storage stability by addition of silk fibroin solution [9,45]. Under optimum conditions, horseradish peroxidase maintained more than 90% activity when stored at 4 °C, room temperature, and 37 °C over 2 months. Using Fourier transform infrared spectroscopy (FT-IR) and differential scanning calorimetry (DSC), they further investigated the mechanism of stabilization of horseradish peroxidase as well as lysozyme in silk fibroin films with respect to β-sheet secondary structure content, water content, and enzyme release through controlling crystallinity by methanol and proteolytic degradation by protease (Figure 7) [72,73]. Moreover, horseradish peroxidase was immobilized via freeze-thaw encapsulation in silk fibroin microspheres (about 2 μm in diameter). The silk fibroin microspheres consisting of physically cross-linked β-sheet structure were prepared using phospholipid vesicles as templates, which were subsequently removed by methanol or sodium chloride treatments (Figure 8). Horseradish peroxidase activity was retained [74].

**Figure 9:**
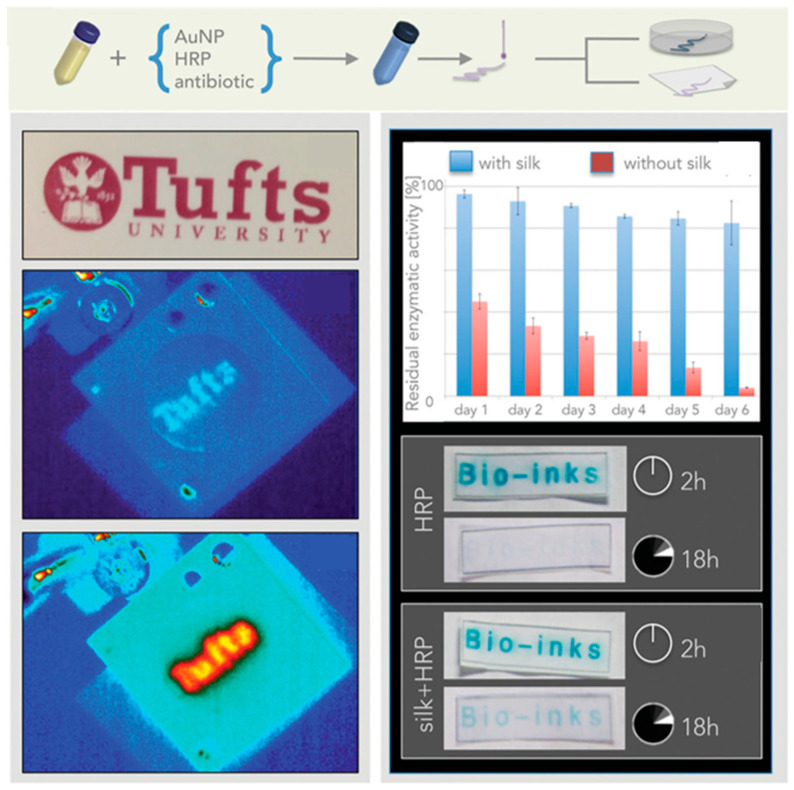
Schematic illustrating the preparation of bioprinting of silk fibroin ink functionalized with horseradish peroxidase (HRP), showing ability of silk to preserve the enzymatic activity. Adapted with permission from REFERENCE [75]. Copyright (2015) John Wiley and Sons.

Omenetto and co-workers investigated bio-printing of silk fibroin (Figure 9). Inkjet-printable water-based horseradish peroxidase-doped silk fibroin inks were printed on conventional paper, and less than 5% loss of horseradish peroxidase was observed with 30 min of the printing process [75]. Horseradish peroxidase activity was also preserved when immobilized via entrapment into silk optical gratings, allowing surface nanopattern down to 125 nm [76,77].

**Figure 10:**
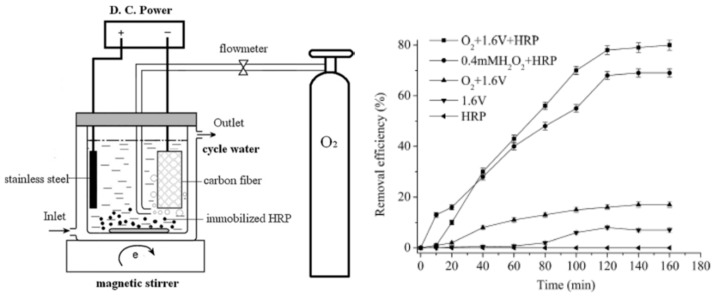
Schematic representation of the experimental apparatus of a membraneless electrochemical reactor based on horseradish peroxidase (HRP) immobilized on silk fibroin nanoparticles, and bisphenol-A removal efficiency during different procedures. Reprinted with permission from REFERENCE [79]. Copyright (2011) Elsevier.

Ai and Zhu reported preparation of a H_2_O_2_ sensor by immobilization of horseradish peroxidase on gold nanoparticle—silk fibroin-modified glassy carbon electrode. A horseradish peroxidase surface coverage of 1.8 × 10^−9^ mol·cm^−2^ was achieved. The immobilized horseradish peroxidase retained 95% residual activity when stored at 4 °C for 30 days [78]. Ai and co-workers investigated immobilization of horseradish peroxidase on silk fibroin nanoparticles for electroenzymatic oxidation of bisphenol-A (organophosphorus compounds) in a membraneless electrochemical reactor (Figure 10). Aminated magnetic silk fibroin nanoparticles were prepared by covalently bond silk fibroin and poly(amido amine) PAMAM onto magnetic Fe_3_O_4_ nanoparticles. Then horseradish peroxidase was covalently immobilized onto the silk fibroin nanoparticles via glutaraldehyde. Under the optimum conditions, 80.3% of bisphenol-A was degraded [79].

### 2.8. Catalase

Hu and co-workers reported immobilization of horseradish peroxidase and catalase in regenerated silk fibroin films on graphite electrodes. The catalase-immobilized silk fibroin films exhibited a pair of well-defined cyclic voltammetric peaks showing potential in development of new biosensors. The immobilized catalase maintained activity in reduction of H_2_O_2_ and nitric oxide [80].

Wang and Fan investigated immobilization of catalase onto ethanol-treated regenerated silk fibroin membranes through physical adsorption and covalent cross-linking to tyrosinase oxidized silk fibroin. The immobilized catalase exhibited higher residual enzyme activity, durability, thermal and pH stability, and alkali resistance than the free catalase [81].

**Figure 11:**
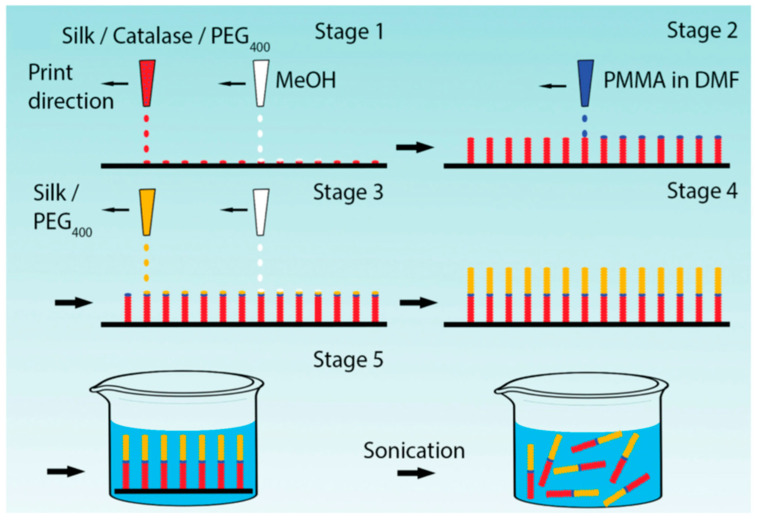
Schematic of Reactive Inkjet Printing (RIJ) process for manufacturing micro-rockets; alternate printing of a silk/catalase/PEG ink and methanol to build the catalytically active half of the micro-rocket on a substrate; printing a PMMA ink to serve as a divider between the two halves of the micro-rocket; alternate printing of a silk/PEG ink and methanol to build the second half of the micro-rocket; immersing the micro-rockets into a fluidic swimming media with ultrasonication to remove from the substrate. Reprinted with permission from REFERENCE [82]. Copyright (2016) John Wiley and Sons.

Zhao and Ebbens reported fabrication of rapidly moving bubble-propulsive self-motile micro-rockets with digitally defined size and shape by alternate inkjet printing of methanol and silk fibroin ink doped with catalase as a propulsion generating enzyme (Figure 11). The enzymatic activity was retained even at acidic pH (pH 4), and the enzyme stability was maintained for long durations [82].

### 2.9. Xanthine Oxidase

Peng and co-workers reported immobilization of xanthine oxidase on a silk fibroin membrane, which together with a cellulose acetate membrane were made into a bilayer coated wire electrode for estimating fish freshness. The resultant electrode was based on detecting H_2_O_2_ released from reaction between xanthine oxidase and hypoxanthine in fish tissue samples, showing high sensitivity, good long-time stability (for 6 weeks or 400 assays) and a detection limit of hypoxanthine of 1 × 10^−7^ M [83].

### 2.10. Tyrosinase

Kundu and co-workers studied covalent immobilization of tyrosinase in silk fibroin fibrous matrix for production of L-DOPA (3,4-dihydroxyphenyl-l-alanine) through bioconversion of tyrosine. Tyrosinase was cross-linked to silk fibroin using glutaraldehyde coupling, leading to an enzyme loading of 50,000 U·g^−1^. The immobilized tyrosinase exhibited high storage (during 10 days), pH (optimum at pH 5.5) and thermal stability (optimum at 40 °C), and reusability, as well as good kinetics/conversion rates with little mass transfer resistances. This study provided a cheaper and more efficient method promising for large-scale production of L-DOPA [84].

Ai and Zhu reported immobilization of tyrosinase on a glassy carbon electrode coated by a composite film of multiwall carbon nanotubes-cobalt phthalocyanine-silk fibroin. Through taking the unique advantages of silk fibroin stabilization effect, the immobilized tyrosinase retained its activity and stability, and was applied in fabrication of an amperometric biosensor for sensitive and reliable determining levels of phenolic compounds, such as assays of bisphenol A in plastic products. With synergistic effect of immobilized tyrosinase, multiwall carbon nanotubes (with excellent inherent electron conductivity) and cobalt phthalocyanine (with good electrocatalytic electrooxidation activity), the biosensor exhibited a well-defined cyclic voltammogram of bisphenol A, a linear correlation between the current signal and the bisphenol A concentration in a range of 5.0 × 10^−8^ to 3.0 × 10^−6^ M with a detection limit of 3.0 × 10^−8^ M under optimum conditions [85].

Li and co-workers reported immobilization of tyrosinase in graphene–silk peptide nanosheets. The immobilized tyrosinase maintained good catalytic activity, and was used in development of an amperometric biosensor for determination of phenolic compounds, based on in situ monitoring of quinine species generated by tyrosinase catalysis of phenolic compounds in the presence of molecular oxygen. The resultant biosensor displayed wide linear range, good sensitivity, repeatability, reproducibility and long-term stability, as well as analytical performance with low detection limits of 0.23, 0.35, and 0.72 nM for catechol, phenol, and bisphenol A, respectively. The biosensor was applied to evaluate trace amounts of bisphenol A leaching from commercial plastic drinking bottles [86].

### 2.11. Acetylcholinesterase

Ai and Zhu reported immobilization of acetylcholinesterase on gold nanoparticles and silk fibroin modified platinum electrodes for pesticide analysis. The immobilized acetylcholinesterase retained its activity, and was applied for development of a simple and inexpensive amperometric biosensor for trace level determination of carbamate and organophosphate pesticides using acetylthiocholine chloride as a substrate. The prepared biosensor showed fast response, high sensitivity, good reproducibility and acceptable stability. Under optimum conditions, the detection limits for methyl paraoxon, carbofuran, and phoxim were estimated to be 2 × 10^−11^ M, 1 × 10^−10^ M, and 2 × 10^−9^ M, respectively [87].

Kang and co-workers reported immobilization of acetylcholinesterase on regenerated silk fibroin by non-covalent adsorption. The acetylcholinesterase-immobilized silk fibroin was coated on a multiwall carbon nanotube-modified glassy carbon electrode, and then developed into an amperometric biosensor for determination of organophosphate and carbamate pesticides using thiocholine as a substrate. The immobilized acetylcholinesterase preserved its activity, and the resultant biosensor showed wide linear ranges, high sensitivity, fast response, well repeatability, acceptable reproducibility, and long-term stability. The detection limits for methyl parathion and carbaryl were found to be 5.0 × 10^−7^ M and 6.0 × 10^−8^ M, respectively. The biosensor was applied to rapidly determine the contents of pesticides in vegetable samples [88].

### 2.12. Neutral Protease

Zhang and co-workers reported covalent immobilization of a neutral protease on silk fibroin nanoparticles. The crystalline silk fibroin nanoparticles were produced by adding an aqueous solution of silk fibroin into excess organic solvent (i.e., acetone). The neutral protease was efficiently cross-linked on silk fibroin nanoparticles using glutaraldehyde, obtaining an enzyme loading ratio of 1 IU per 58 mg silk fibroin. The immobilized neutral protease showed improved thermal and pH stability and activity in vitro, and was used repeatedly to hydrolyze silk sericin into sericin peptides. The range of molecular masses of the sericin peptide produced (<30 kDa) could be adjusted by extending the reaction time. This study provided an inexpensive method for large-scale production of sericin peptides, showing great potential for practical applications in food processing. In addition, this method was further employed for immobilization of L-asparaginase and β-glucosidase [89].

### 2.13. α-Chymotrypsin

Lee and co-workers reported immobilization of α-chymotrypsin onto electrospun silk fibroin fibers. Silk fibroin fibers were modified with silk sericin using glutaraldehyde. The immobilized α-chymotrypsin exhibited good stability against denaturation, retaining 78% activity even after 1 h of ethanol treatment [90]. Moreover, silk fibroin nanofibers with different diameters were prepared by electrospinning, showing high enzyme loading up to 5.6 wt%. The immobilized α-chymotrypsin on silk fibroin nanofiber exhibited higher stability and activity, which was eight times of than that on silk fibroin fiber, and increased along with decrease in the fiber diameter [91]. Furthermore, two different operation modes for the α-chymotrypsin-immobilized electrospun silk fibroin nanofibrous membrane were compared, showing lower Michaelis–Menten K_m_ and higher V_max_ in the membrane reactor mode than that in the batch reactor mode [92].

### 2.14. Amylase

Rani et al. reported covalent immobilization of amylase on woven silk fibroin fabric through glutaraldehyde coupling. The woven fabric was chemically charged by chlorination and diazotization activation. Immobilization improved enzyme thermal stability; the optimum temperature increased to 60 °C as compared to 50 °C of the free enzyme. The immobilized amylase remained stable for more than 4–5 months when stored at 4 °C in 1 M KCl solution. These results suggested a potential method for immobilization of amylase in food and pharmaceutical applications [93].

### 2.15. Organophosphorus Hydrolase/Aryldialkylphosphatase

Naik and co-workers studied immobilization of organophosphorus hydrolase in silk fibroin through entrapment. Silk fibroin was dissolved in a neutral pH salt solution and dialyzed against water. An aqueous solution of the regenerated silk fibroin was mixed with organophosphorus hydrolase and subsequently dried or gelled. Immobilization preserved organophosphate hydrolysis activity and increased stability of organophosphorus hydrolase under a variety of harsh environmental conditions, such as thermal denaturation, UV light exposure, detergents, and organic solvents. Silk fibroin entrapment allowed more complex formulations of organophosphorus hydrolase, such as dispersal into a polyurethane-based coating, promising for bioremediation of organophosphate insecticides on an industrial scale and chemical warfare agents [94].

### 2.16. β-Galactosidase

Monier studied covalent immobilization of β-galactosidase on polyacrylonitrile-grafted natural worm silk fibers. The modified silk fibers were prepared by graft copolymerization of polyacrylonitrile in the presence of benzophenone as a photoinitiator, and then activated by hydrazine hydrate. β-galactosidase was cross-linked by glyoxal. After immobilization, Michaelis–Menten constant K_m_ increased, V_max_ decreased, the optimum pH shifted slightly to 7 as compared to 6.5 of the free enzyme, and the optimum temperature increased by 5 °C, suggesting improved pH and thermal stability [95].

### 2.17. Carbonic Anhydrase

Barralet and co-workers reported immobilization of carbonic anhydrase on silk fibroin-coated hydroxyapatite microparticles through ultrasonically bonded entrapment. The immobilized carbonic anhydrase exhibited a remarkable operational and storage stability. Particularly, immobilized carbonic anhydrase maintained almost 100% activity after 1 h at 110 °C, and 45% activity after 3 weeks at 80 °C in an amine solution, indicating excellent thermal stability over that of the free enzyme [96].

**Figure 12:**
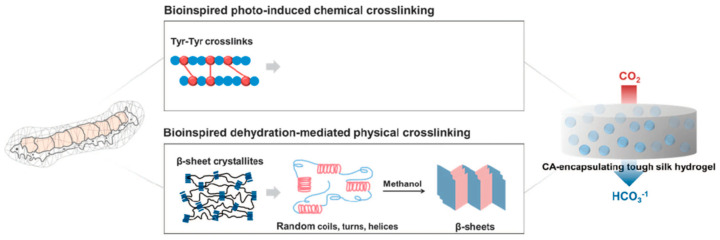
Schematic illustration of proposed mechanism of carbonic anhydrase (CA)-encapsulating silk hydrogel for CO_2_ sequestration. Reprinted with permission from REFERENCE [97]. Copyright (2017) Springer Nature.

Cha and co-workers reported covalent immobilization of carbonic anhydrase in silk fibroin-based hydrogels through photo-induced dityrosine chemical cross-linking followed by dehydration-mediated physical cross-linking via formation of β-sheet structures (Figure 12). The immobilized carbonic anhydrase retained ~60% activity, storage and thermal stability. In addition, the immobilized carbonic anhydrase maintained ~97% activity after 6 repeated cycles. The carbonic anhydrase-immobilized silk fibroin-based hydrogels could be used as a robust biocatalyst for environment-friendly sequestration of carbon dioxide under mild conditions to produce value-added chemicals (including calcium carbonate) [97].

**Figure 13:**
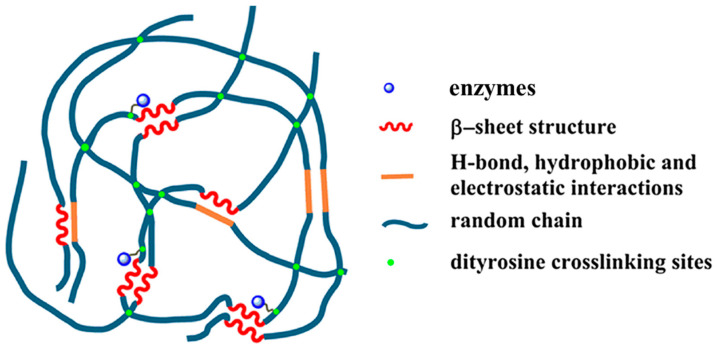
Schematic representation of proposed network of silk fibroin-based hydrogel immobilization and stabilization of enzymes. Adapted with permission from REFERENCE [98]. Copyright (2018) Elsevier.

Our group fabricated high water content silk fibroin-based hydrogels with tunable elasticity through Ru(II)-mediated photo-chemical cross-linking of tyrosine residues [137,138,139]. The resultant hydrogels exhibited a good performance as efficient and effective carriers for immobilization of carbonic anhydrase, xylanase, and lysozyme against pH denaturation (Figure 13). The immobilized carbonic anhydrase not only retained >60% activity for p-nitrophenyl acetate (p-NPA) hydrolysis at the optimum pH value of 8, but also showed activity at unfavorable acidic pH values down to 3, as compared to complete deactivation of the free enzyme under the same experimental conditions. In addition, immobilization enabled recyclability. The immobilized xylanase and lysozyme achieved better activity at an unfavorable basic pH value of 9. This study provides insight into the silk fibroin-based hydrogel approach for the promising applications in fairly simple and straightforward enzyme immobilization [98].

### 2.18. Laccase

**Figure 14:**
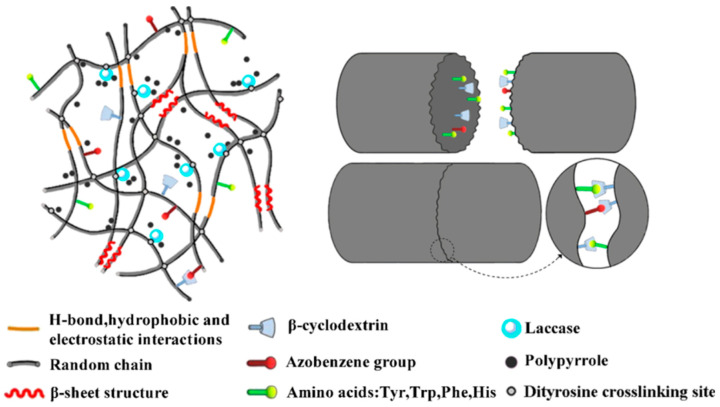
Schematic representation of proposed network of silk fibroin-based hydrogel immobilization of laccase for enzymatic polymerization of pyrrole. Reprinted with permission from REFERENCE [99]. Copyright (2019) American Chemical Society.

**Figure 15:**
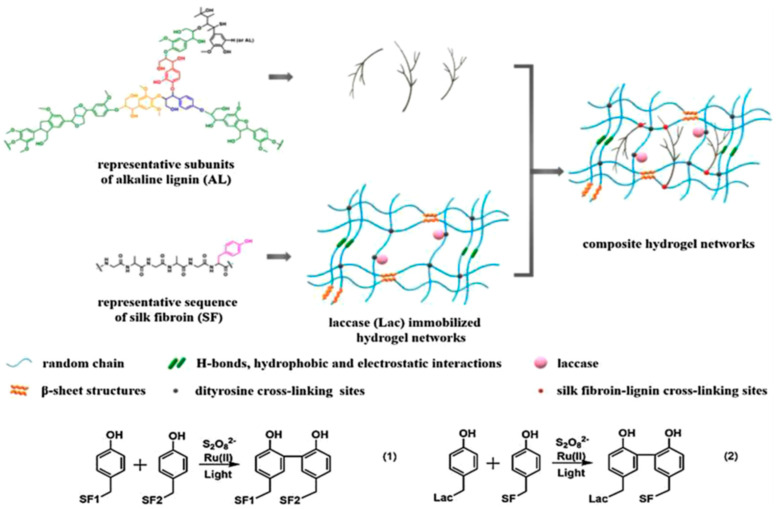
Schematic illustration of proposed network of silk fibroin (SF)-lignin composite hydrogels prepared by immobilized laccase (Lac) catalyzed grafting of lignin. Both the SF-SF cross-linking sites and the SF-Lac cross-linking sites were dityrosine cross-linking sites shown as black dots; the SF-lignin cross-linking sites were shown as red dots. Reprinted with permission from REFERENCE [100]. Copyright (2020) Elsevier.

Our group extended the above-mentioned Ru(II)-mediated photo-chemical cross-linked silk fibroin-based hydrogels to immobilization of laccase. The immobilized laccase was employed for in situ polymerization of pyrrole using the as-prepared hydrogels as molecular templates (Figure 14), giving rise to polypyrrole-coated hydrogels with an electrical conductivity of (1.0 ± 0.3) × 10^−3^ S·cm^−1^ [99]. Moreover, the laccase-immobilized silk fibroin-based hydrogels were used for fabrication of regenerated silk fibroin-alkaline lignin composite hydrogels (Figure 15). The immobilized laccase retained activity in efficiently catalyzing inter- and intra-molecular covalent coupling between phenolic groups of alkaline lignin and tyrosine residues of silk fibroin using ambient air. The resultant composite hydrogels displayed enhanced mechanical strength and anti-ultraviolet properties, representing a new type of sustainable value-added materials based on silk and lignin. In addition, this study demonstrated that diffusional limitations encountered in application of immobilized enzymes including physical adsorption of reactant and trapping of product could be turned into advantages and making good use for constructing composite hydrogels [100].

### 2.19. Zymolyase

Our group further studied immobilization of the lytic enzyme zymolyase on Fe_3_O_4_-embedded silk fibroin magnetic microspheres, which were prepared by solvent (ethanol)-induced self-assembly of silk fibroin surrounding Fe_3_O_4_ magnetic nanoparticles, presynthesized by a co-precipitation method. Then zymolyase was covalently attached onto surface of the magnetic microspheres by the above-mentioned Ru(II)-mediated photochemical cross-linking method with high immobilization efficiency, showing an enzyme loading capacity of 100 mg·g^−1^. The immobilized zymolyase exhibited good activity and stability for disruption of S. cerevisiae cells in a wide range of pH. At unfavorable acidic pH of 4, the immobilized zymolyase retained 81% activity as compared to complete deactivation of the free enzyme. In addition, the zymolyase-immobilized microspheres showed a saturation magnetization value of 53.8 Gs, enabling recyclability of the immobilized zymolyase by simple magnetic separation. The study validated silk fibroin magnetic microspheres as promising enzyme immobilization platforms with superior performance [101,140].

### 2.20. L-Asparaginase

Shen and co-workers studied covalent immobilization of L-asparaginase on silk fibroin powder by glutaraldehyde. The immobilized L-asparaginase showed increased the enzyme substrate affinity (a K_m_ value of 0.844 × 10^−3^ mol·L^−1^ approximately 6 times lower than that of the free enzyme) and circulating half-life (63 h longer than 33 h of the free enzyme), improved thermal and pH stability, resistance to trypsin digestion, and storage stability (with 80% activity retained after 30 days at room temperature) [102]. Moreover, Zhang et al. reported immobilization of L-asparaginase on silk fibroin nanoparticles by adding a mixture of regenerated silk fibroin and L-asparaginase into excess acetone rapidly. L-asparaginase was efficiently immobilized in the silk fibroin nanoparticles, with no observation of enzyme leaching. The L-asparaginase-immobilized silk fibroin nanoparticles were 50–120 nm in diameter, with 90% activity and similar Michaelis–Menten kinetics as compared to the free enzyme [103]. Considering that L-asparaginase is a drug effective in treatment of acute lymphoblastic leukemia, silk fibroin immobilization of L-asparaginase enabled potential use in practical clinic. In addition, this method could be extended to immobilization of other therapeutic enzymes, such as 𝛽-glucosidase [104]. Wang and co-workers studied optimization of L-asparaginase immobilization on silk fibroins. The optimum conditions were found to be an enzyme loading of ~16 wt%, a temperature of 4 °C, a pH of 7.0 and a period of 8 h [105].

### 2.21. Phenylalanine Ammonia-Lyase

Inoue et al. studied immobilization of phenylalanine ammonia-lyase in silk fibroin microparticles by encapsulation. The silk fibroin microparticles were ~150 μm in diameter. The immobilized phenylalanine ammonia-lyase showed enhanced thermal and storage stability, with 75.4% activity retained after storage at 48 °C for 82 days as compared to 34.4% of the free enzyme. The immobilized phenylalanine ammonia-lyase also showed resistance to chymotrypsin and trypsin proteolysis, allowing retaining activity in the intestinal tract following oral administration, which was proved by in vivo experiments in rat duodenum using cinnamate as a model. Silk fibroin immobilization of phenylalanine ammonia-lyase provided a promising oral enzyme therapy of phenylketonuria [106].

### 2.22. Thymidine Kinase

Ghandehari and co-workers studied immobilization of herpes simplex virus thymidine kinase (HSVtk)-ganciclovir (GCV) system in recombinant silk-elastin-like protein polymers. The recombinant silk-elastin-like protein polymers, which are composed of tandem repeats of a six amino acid sequence commonly found in silk fibroin (GAGAGS) and a five amino acid sequence commonly found in elastin (GVGVP), irreversibly form hydrogel networks at 37 °C based on β-sheet formation of the silk-like sequences. Due to swelling properties, the hydrogels have been investigated for viral gene delivery in anticancer treatment using virus-mediated gene directed enzyme prodrug therapy. The efficacy of the HSVtk-GCV system for head and neck cancer gene therapy was significantly improved by immobilization in the recombinant silk-elastin-like protein polymers [107]. Recombinant silk-elastin-like protein polymers were also found to improve the anticancer activity using adenoviral-directed enzyme prodrug therapy for intratumoral viral delivery of thymidine kinase-1 and luciferase genes, offering a promising approach for head and neck cancer gene therapy [108].

## 3. Immobilization of Non-Enzymatic Catalysts

### 3.1. Gold (Au)

In 1956, Akabori and co-workers reported silk proteins as supports of zero-valent metal catalysts for asymmetric hydrogenation [141,142,143,144]. However, since then few advances have been noted. Recently, Naka and Chujo reported preparation of a highly monodispersed core-shell nanostructured gold colloid–silk fibroin bioconjugate through in situ reduction of Au(III) ions by the tyrosine residues of silk fibroin. Gold colloid-silk fibroin bioconjugates with an average size of 45 nm and gold nanoparticle cores with an average size of 15 nm were obtained. The bioconjugate solution rendered high stability during storage in air at room temperature for more than three months, providing promising protein-metal colloid conjugates for application in biotechnology, biochemistry and medicine [109].

**Figure 16:**
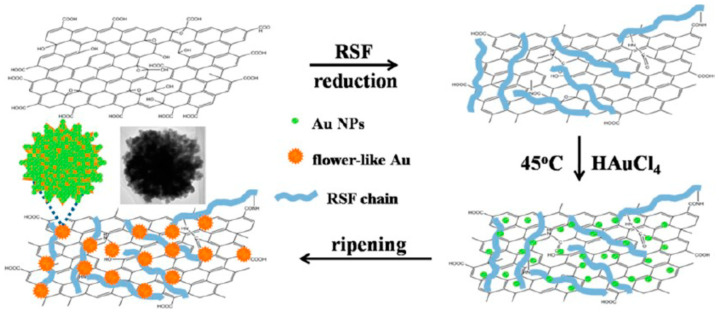
Schematic illustration of procedure for silk fibroin-assisted synthesis of flowerlike composites of Au nanoparticles and reduced graphene oxide. Reprinted with permission from REFERENCE [110]. Copyright (2013) American Chemical Society.

Wu and co-workers studied a flowerlike composite of Au nanoparticles and reduced graphene oxide. The composite was prepared in a facile, rapid and green approach in the presence of regenerated silk fibroin (Figure 16). Regenerated silk fibroin easily and tightly absorbed onto reduced graphene oxide surfaces by π-π stacking and H-bonding, and provided nucleation sites for binding Au nanoparticles, thanks to its amino acid composition such as glycine, alanine, serine, and tyrosine. The composite could be redispersed in water stably. The composite showed improved catalytic activity toward ORR in electrochemistry, as well as absorption throughout visible and near-infrared region in colorimetric sensing [110].

**Figure 17:**
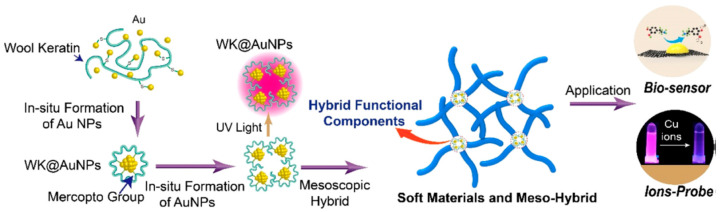
Schematic illustrations of preparation process and hierarchical structures of SF-WK@AuNP mesoscopic hybrid materials, and application as electrochemical sensors. Reprinted with permission from REFERENCE [111]. Copyright (2019) American Chemical Society.

Liu and co-workers studied mesoscopic construction of wool keratin-Au nanoparticle–silk fibroin hybrid materials (Figure 17). Wool keratin was introduced to in-line synthesize Au nanoparticles, because of high content of mercapto group (-SH). The wool keratin-Au nanoparticle–silk fibroin hybrid materials showed extraordinary fluorescence emission with long-term stability and high-intensity, which could be applied for rapid detection of copper ions (Cu^2+^) in drinking water with high sensitivity and selectivity. After carbonization, the wool keratin-Au nanoparticles–silk fibroin hybrid materials turned into secondary hybrid materials of carbon-Au with good electrical conductivity, which could be used in fabrication of high-performance electrochemical sensors for dopamine [111].

Zhao and co-workers studied loading of Au nanozyme stabilized by bovine serum albumin using silk fibroin hydrogels as carriers, leading to a solid-state biocompatible sensor for visual detection of H_2_O_2_ by fluorescence quenching. The sensor showed fast response, good stability and high sensitivity with a detection limit of 0.072 mM. Considering that H_2_O_2_ is an important biological indicator, the Au nanozyme-silk fibroin hybrid hydrogels showed great potential for in vivo continuous H_2_O_2_ monitoring in clinical diagnosis of diseases [112].

**Figure 18:**
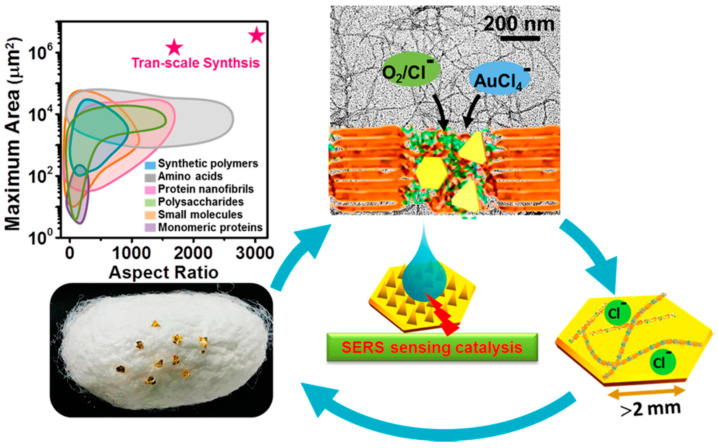
Schematic illustration of preparation of millimetric 2D Au crystals with silk nanofibrils. Reprinted with permission from REFERENCE [113]. Copyright (2018) American Chemical Society.

Mezzenga and Li studied production of millimetric large Au single-crystals in the presence of silk fibroin (Figure 18). Due to its unique amino acid sequence and supramolecular assembly architectures, silk fibroin has mild reducing and strong capping effects. Together with Cl^−^, two-dimensional (2D) Au single-crystals with an unprecedented lateral length of ~2.4 mm and a planar area of ~3.4 mm^2^ were successfully synthesized. These Au single-crystals represented unique platforms for catalysis, sensing, and optoelectronics applicable in nanotechnology, biomedicine, and environments [113].

### 3.2. Palladium (Pd)

**Figure 19:**
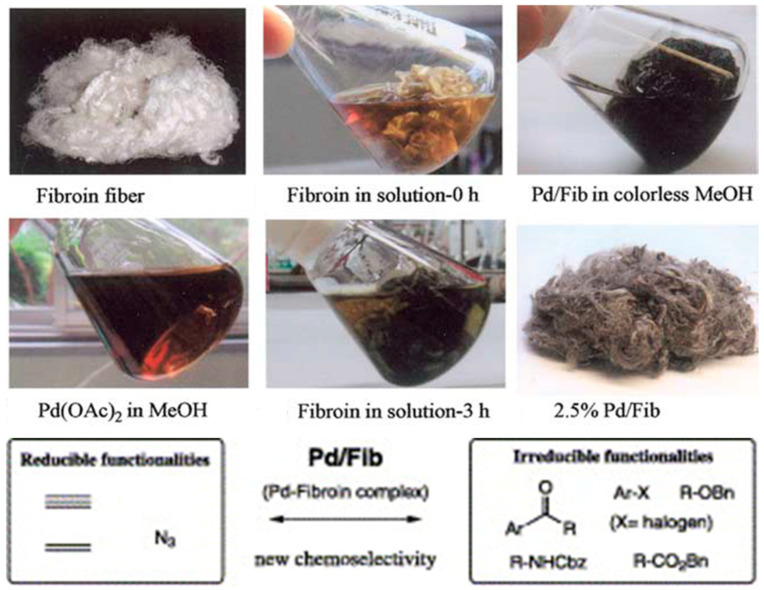
Preparation of silk fibroin supported palladium (Pd/Fibroin) catalyst for chemoselective hydrogenation. Reprinted with permission from REFERENCE [116]. Copyright (2005) Elsevier.

Sajiki and Hirota reported silk fibroin supported zero-valent palladium Pd(0) catalyst for chemoselective hydrogenation (Figure 19). The catalyst was prepared by reduction of silk fibroin conjugated palladium(II) acetate Pd(OAc)_2_ by methanol and/or silk fibroin at room temperature in air. The catalyst displayed highly dispersed Pd within 1–10 wt% of silk fibroin, and good chemoselectivity in heterogeneous phase hydrogenation of olefins and azides in the presence of aromatic carbonyls and halogens or an O-benzyl protective group. In addition, the catalyst could be easily manipulated (cutting by scissors and separation by tweezers or simple filtration), and exhibited stability over a year at room temperature [114,115,116].

### 3.3. Iron (Fe)

Bayraktar and co-workers studied use of silk fibroin fibers as supports of iron catalyst for phenol hydroxylation reactions. The catalyst was prepared by a simple method using formic acid at room temperature with no observation of significant iron leaching. The catalyst was flexible and easy-handling, and exhibited excellent activity in hydroxylation of phenol to dihydroxybenzenes (catechol and hydroquinone) using hydrogen peroxide as an oxidant, achieving phenol conversions of 3.3%, 61.2%, and 80.3% at 25 °C, 40 °C, and 60 °C, respectively. In addition, the catalyst was reusable for three repeated cycles without significant decrease in activity [117].

**Figure 20:**
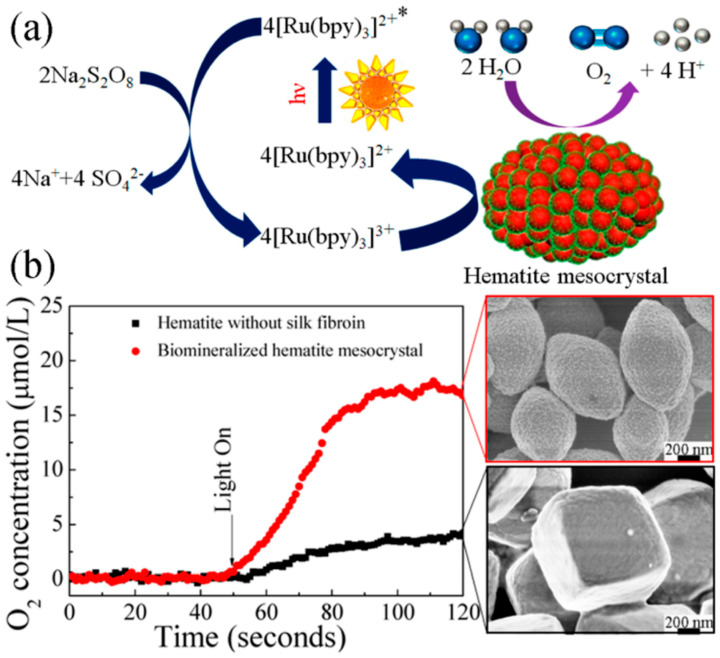
Schematic presentation of α-Fe_2_O_3_ biomineralized hematite mesocrystal prepared in the presence of silk fibroin, and its application as catalyst in photocatalytic water oxidation. (**a**) Proposed reaction mechanism using Na_2_S_2_O_8_ as sacrificial electron acceptor, and [Ru(bpy)_3_]^2+^ as photosensitizer. (**b**) Time courses of O_2_ evolution under photo irradiation (λ = 470 nm) at room temperature. Reprinted with permission from REFERENCE [119]. Copyright (2014) American Chemical Society.

Chen and co-workers reported silk fibroin supported magnetic hematite (α-Fe_2_O_3_) nanostructures by a hydrothermal method. The resultant α-Fe_2_O_3_ showed uniformly monodispersed morphologies with fine size and shape control by varying silk fibroin concentration, including quasi-nanocubes, nanospheres, and nanoparticles [118]. In addition, hematite mesocrystals with uniform porous nanostructures and controllable morphologies were synthesized through a biomineralization process using silk fibroin as a biotemplate (Figure 20). The synthesized α-Fe_2_O_3_ exhibited good photocatalytic performance for oxygen evolution via water oxidation in the visible-light-driven [Ru(bpy)_3_]^2+^-persulfate system. These results provided an efficient and green approach for large-scale production of functional mesocrystals, promising in energetic and environmental research fields [119].

Shao and co-workers used regenerated silk fibroin-based hydrogels with 10% hydroxypropyl methylcellulose for simple and facile in situ synthesis of magnetic ferriferous oxide (Fe_3_O_4_) via co-precipitation of Fe^2+^ and Fe^3+^. The resultant magnetic hydrogels exhibited excellent peroxidase-like catalytic activity in 3,3′,5,5′-tetramethylbenzidine (TMB)-mediated detection of H_2_O_2_ with a low limit of 1 × 10^−6^ mol⋅L^−1^. In addition, the low-cost catalyst showed long-term stability under various conditions, promising for environmental chemistry and biotechnology applications [120].

### 3.4. Titanium Dioxide (TiO_2_)

**Figure 21:**
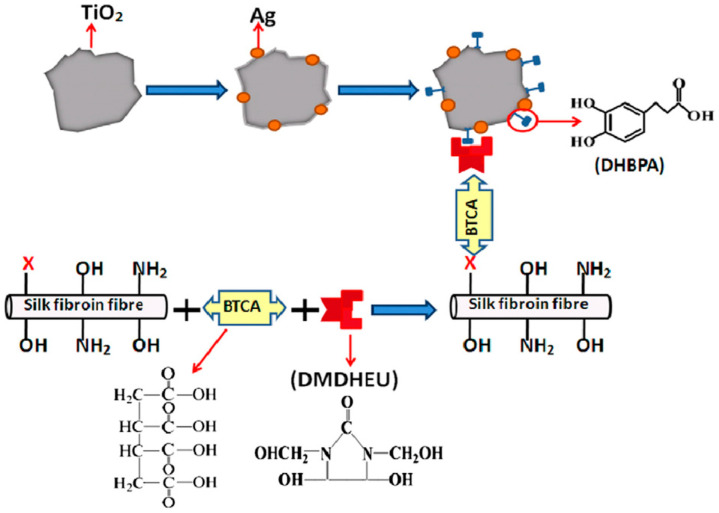
Schematic illustration of TiO_2_@Ag nanoparticles covalently bonded onto pre-modified silk fibroin fiber surface. Reprinted with permission from REFERENCE [121]. Copyright (2011) Elsevier.

Liu and co-workers reported densely and uniformly assembly of TiO_2_ and TiO_2_@Ag nanoparticles on silk fibroin fabric via enediol ligand–metal oxide bonding through a pad-dry-cure and durable-press treatment process (Figure 21). The nanoparticles were modified by 3-(3,4-dihydroxyphenyl) propionic acid (DHBPA); the fabric was treated with dimethyloldihydroxyethyleneurea (DMDHEU) resin using 1,2,3,4-butanetetracarboxylic acid (BTCA) with the assistance of sodium hypophosphite (NaH_2_PO_2_); the carboxylic acid groups of DHBPA reacted with the hydroxyl groups of DMDHEU, covalently cross-linking the nanoparticles onto the fabric. The TiO_2_- and TiO_2_@Ag-loaded silk fibers exhibited high photocatalytic activity in photodegradation of methylene orange under UV illumination [121].

Chang and co-workers studied co-deposition of anatase TiO_2_ and amorphous Ni-P metallization layer on silk textile via supercritical carbon dioxide promoted electroless plating to fabricate a flexible composite toward wearable devices. The resultant silk textile was endowed with electrically conductivity of Ni-P and photocatalytic activity of TiO_2_ with a critical concentration of 30 g·L^−1^ [122].

### 3.5. Platinum (Pt)

Tang and Wang reported convenient and facile in situ synthesis of platinum nanoparticles on silk fabric through reduction of platinum ions by heat treatment. The effect of ion concentration, pH, and temperature was systematically investigated, showing that low acidic condition and high temperature were conducive to formation of platinum nanoparticles. The platinum substituted silk fabric exhibited excellent catalytic activity in conversion of 4-nitrophenol into 4-aminophenol [123].

**Figure 22:**
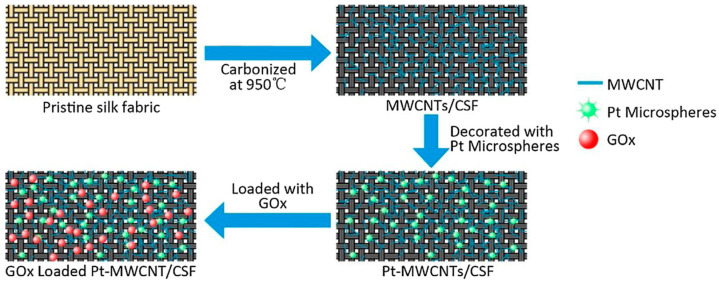
Schematic illustration of preparation process of glucose sensors based on glucose oxidase (GOx)-immobilized platinum microspheres (Pt) and multiwalled carbon nanotube (MWCNT)-coated carbonized silk fabric silk fabric (CSF). Reprinted with permission from REFERENCE [124]. Copyright (2018) Elsevier.

Ran and Huang studied electrochemical deposition of platinum microspheres on multi-walled carbon nanotubes coated carbonized silk fabric, which was prepared by carbonization at 950 °C under an inert atmosphere (Figure 22). The resultant material possessed good electrical conductivity, and could be used to fabricate electrodes with a good sensitivity towards electrochemical detection of H_2_O_2_. After further immobilization of glucose oxidase through immersing into a mixture of glucose oxidase and Nafion, an efficient flexible electrochemical glucose sensor with high sensitivity and good stability could be obtained. This study provided a simple method for design of wearable electronic devices [124].

### 3.6. Zinc Oxide (ZnO)

**Figure 23:**
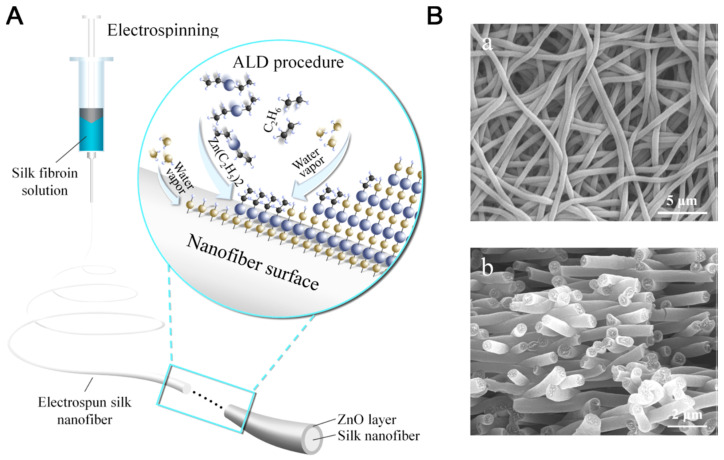
Schematic illustration of preparation of silk/ZnO silk/ZnO nanofibers. (**A**) Fabrication of electrospun silk nanofibers and surface modification by layer-by-layer deposition of ZnO through ALD. (**B**) SEM images for (**a**) silk and (**b**) silk/ZnO materials. Reprinted with permission from REFERENCE [125]. Copyright (2017) American Chemical Society.

Zhang and co-workers studied deposition of metal oxide layers on electrospun silk fibroin nanofibers through atomic layer deposition (ALD), using ZnO as a model considering its wide applications in antibacterial, optical, and sensing (Figure 23). The ZnO layer displayed hexagonal wurtzite structure, excellent uniformity and 3D conformity, and exhibited temperature-dependent photocatalytic activity in photodegradation of rhodamine B under UV exposure. This study provided an easy, efficient, and controllable method for fabrication of multifunctional organic/metal oxide composite biomaterials [125].

**Figure 24:**
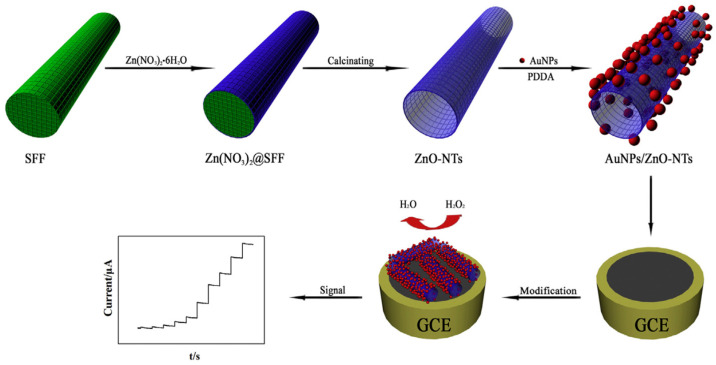
Schematic presentation of fabrication process of the non-enzymatic H_2_O_2_ sensor based on silk fibroin fibers modified with AuNPs/ZnO-NTs. Reprinted with permission from REFERENCE [126]. Copyright (2018) Elsevier.

Chen and Xu reported synthesis of a composite of Au nanoparticles and ZnO nanotubes in the presence of silk fibroin fibers (Figure 24). The ZnO nanotubes were prepared by a convenient biomineralization strategy using natural silk fibroin fibers extracted from silkworm cocoons as templates for anchoring zinc nitrate Zn(NO_3_)_2_. During calcination, the silk fibroin fiber template was removed, and Zn(NO_3_)_2_ decomposed into ZnO forming nanotubes. Then, Au nanoparticles were coated on the surface of the ZnO nanotubes by electrostatic absorption. The resultant composites of Au nanoparticles-ZnO nanotubes showed catalytic activity in reduction of H_2_O_2_, and thereby used to construct an electrochemical sensor for non-enzymatic detection of H_2_O_2_. The sensor exhibited high sensitivity and selectivity with a detection limit of 0.1 μM [126].

Chen and co-workers reported facile fabrication of ZnO/Au layered structure on silk textiles. Smooth and uniform coverage of Au metallic layer on silk was achieved by supercritical carbon dioxide promoted electroless plating; then wurtzite ZnO was homogeneously coated on the Au metallized silk by cathodic deposition. The resultant composite displayed high electrically conductive and photocatalytic performance, promising for flexible and wearable devices such as in solar energy harvesting applications [127].

### 3.7. Cupric Oxide (CuO) and Cuprous Oxide (Cu_2_O) Nanoparticles

**Figure 25:**
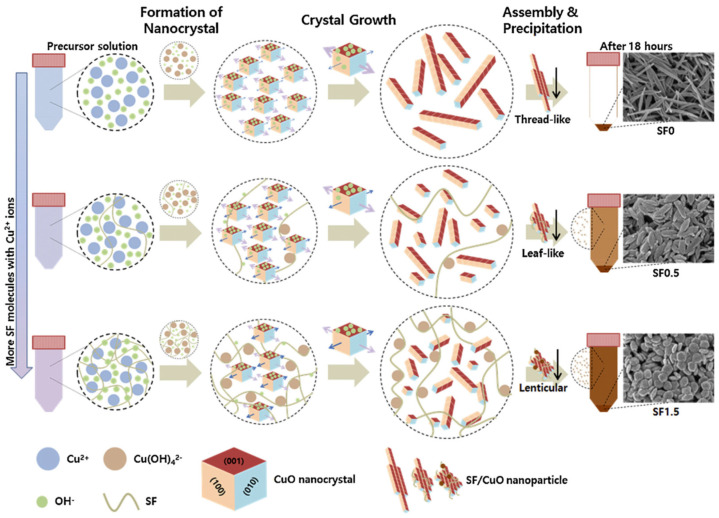
Schematic illustration of possible mechanism of SF/CuO nanoparticle formation. Reprinted with permission from REFERENCE [128]. Copyright (2017) Elsevier.

Park and co-workers reported facile synthesis of uniform CuO nanoparticles using silk fibroin as template through a simple wet chemical method from a precursor aqueous solution containing Cu^2+^ copper(II) acetate monohydrate and silk fibroin in the presence of sodium dodecyl sulfate under alkali condition (Figure 25). The CuO crystal structure, morphology, shape, size, and surface properties of the nanoparticles could be controlled by tuning the amount of silk fibroin in the nanoparticles. The silk fibroin/CuO hybrid mesoporous nanoparticles showed excellent photocatalytic performance in photodegradation of Congo Red (128.30 mg·g^−1^ with 0.1% silk fibroin). These results showed great potential in applications of photocatalytic purification of sewage [128].

Zhang and co-workers reported direct synthesis of core–sheath structured Cu_2_O nanoparticles embedded in carbon spheres on carbonized silk fabrics. The materials were prepared by loading Cu-BTC on silk fabric followed by carbonization, resulting in flexible and soft carbon cloth with good electrical conductivity. When fabricated into an electrode, the resultant non-enzymatic glucose sensor showed superior electrochemical performance, in terms of high sensitivity, selectivity, and stability, as well as a low detection limit of 0.29 μM. These results offered a promising low price flexible yet self-supported electronic device for detection of blood sugar levels in practical applications [129].

### 3.8. Trimanganese Tetraoxide (Mn_3_O_4_) and Manganese Dioxide (MnO_2_) Nanoparticles

Morsali and co-workers reported silk yarn coated with sphere-like trimanganese tetraoxide (Mn_3_O_4_) nanoparticles by a simple sonochemical method via sequential dipping in potassium hydroxide and manganese(II) nitrate alternatively under ultrasound. Presence and abundance of Mn element on silk yarn was confirmed by powder X-ray diffraction (XRD) and wavelength dispersive X-ray (WDX) characterization. Scanning electron microscopy (SEM) analysis showed that size of the Mn_3_O_4_ nanoparticles decreased along with decrease in pH [130].

Shaabani and co-workers reported in situ heterogeneous production of manganese dioxide (MnO_2_) nanostructures on natural silk fibers by simple immersion in an aqueous solution of permanganate (KMnO_4_), where the silk fibers served as a reducing agent. The MnO_2_ coating exhibited high catalytic activity, selectivity, and recyclability in aerobic oxidation (using alkyl arenes, alcohols, and oximes as models), and tandem oxidative synthesis of organic compounds (using a one-pot two-component reaction of aromatic hydrocarbons of petroleum naphtha as a model) [131].

**Figure 26:**
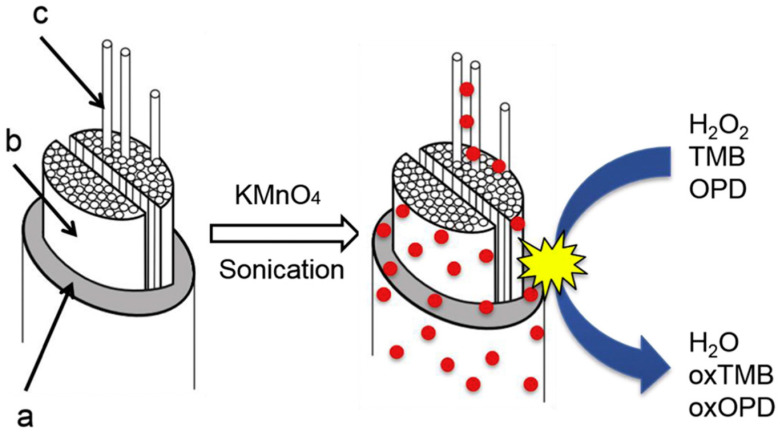
A proposed mechanism for MnO_2_ formation and deposition on and into the silk fiber upon sonication. The fiber to the left is the native silk with sericin coating (a), thefibroin brins (b),and thefibrils (c). The stabilized MnO_2_-silk hybrid showed catalytic activities like catalase, oxidase, and peroxidase. Reprinted with permission from REFERENCE [133]. Copyright (2020) Elsevier.

Singh and Dicko reported in situ synthesis and stabilization of MnO_2_ nanoparticles on four different silk yarns (i.e., mulberry, tasar, muga, and eri silks). The resultant hybrids displayed good performance in catalytic oxidation of dyes (such as methylene blue) [132]. They further studied the catalytic origin of silk stabilized MnO_2_ nanoparticles through evaluation of their catalase, oxidase, and peroxidase-like activities using H_2_O_2_, 3,3′,5,5′-tetramethylbenzidine (TMB), O-phenylenediamine as substrates (Figure 26). It was found that the MnO_2_-silk hybrid could be used as oxidoreductase enzyme mimics [133]. Moreover, sonication (using 3 mm probe sonicator, 30 W, 20 kHz in the presence of 0.5 mM of KMnO_4_ at 20–24 °C) was found to enhance stability of smaller and more monodispersed MnO_2_ nanoparticles on silk films for the enzyme mimic application, such as achieving higher catalytic activity and stability with 3,3′,5,5′-tetramethylbenzidine (TMB) substrate [134]. In addition, a polypyrrole-MnO_2_-silk hybrid prepared by combined supercritical carbon dioxide impregnation of pyrrole and sonochemical transformation of KMnO_4_ on silk fibers was reported. The prepared hybrid showed independent and complementary conductivity and enzyme-like catalytic activities in degradation of H_2_O_2_, and thereby could be fabricated into soft electrodes and sensors for detection of H_2_O_2_ [135].

## 4. Summary and Outlook

Silk fibroin-based materials have been found to be excellent stabilizing carries/supports for immobilization of catalysts, including both enzymes and non-enzymatic catalysts. An advantage in using silk fibroin is its capability of both adsorption/entrapment of catalysts by physical or mechanical treatment and covalent coupling of catalysts by chemical treatment. Silk fibroin in various forms of gel, powder, fiber, and membrane has been prepared depending on the explored applications ranging from chemicals, pharmaceuticals, food, agriculture, energy, environment, and pulp and paper industry [25,26,32,39,40,59]. However, there are still challenges and further needs for commercializing silk fibroin-stabilized enzymes and silk fibroin-supported metal catalysts as cheap and sustainable catalysts.

Three possible directions are suggested for future research and development: (1) industrially important enzymes, (2) therapeutic enzymes, and (3) metal/metal oxide catalysts. For example, lipase and amylase are two important industrial enzymes in food market, in which more ‘‘green’’ enzymes/biocatalysts are favored. In addition to food and cosmetics, diagnostic and therapeutic applications also favor more “safe” enzymes/biocatalysts and biocompatible immobilization materials. Because catalytic activity of enzymes and metal/metal oxide tends to be lost as a result of chemical modifications, conformational change, degradation, aggregation, and leakage during preparation, operation and storage, the challenge in stabilization of catalysts using the silk fibroin-based approach remains selecting appropriate immobilization methods and rational design strategies [13].

**Figure 27:**
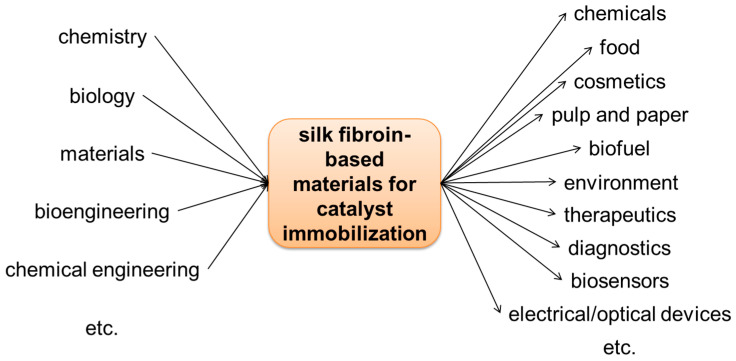
Interdisciplinary research in silk fibroin-based materials for catalyst immobilization for potential applications in different industries.

The research of silk fibroin-based materials for catalyst immobilization is an interdisciplinary area, overlapping chemistry, biology, materials, chemical engineering and bioengineering (Figure 27). For example, chemists can contribute on catalyst synthesis and analysis, and catalysis mechanism clarification. Biologists can contribute on protein engineering, directed evolution, and enzyme characterization. In particular, immobilized catalysts encounter problems such as diffusion limitations, chemical engineers can contribute in providing solutions in terms of mass transfer, heat transfer, momentum transfer, and chemical reaction process. Researchers from different disciplines may need to collaborate to address the challenges for successful practice of silk fibroin-based catalyst immobilization.

## Figures and Tables

**Table 1:** Silk fibroin-stabilized enzymes/biocatalysts listed in this review.

Section	Immobilized Enzymes/Biocatalysts	Forms	Methods	Explored Applications	References
2.1	alkaline phosphatase	woven silk	absorption and covalent bond through acid methylation, glutaraldehyde and azide/diazo-coupling		[7,8,16]
	*aspartate aminotransferase* ^a^				
	*ribonuclease A* ^a^				[16]
	*rennet* ^a^				
	*glycyl-tRNA-synthetase* ^a^				
		fibers	covalent bond through diazo and cyanogen bromide coupling		[17,18]
		scaffolds	entrappment	calcium phosphate mineralization	[19]
2.2	β-glucosidase	membranes	entrappment		[20]
		eri silk fibrion microparticles	adsorption	cellobiose hydrolysis	[21]
	*naringinase (* *a bienzyme system of* *𝛼* *-l-rhamnosidase* *and* *flavonoid-* *𝛽* *-glucosidase)* ^a^	nanoparticles	glutaraldehyde	juice debittering	[22]
2.3	glucose oxidase	membranes	entrapment and glutraldehyde		[23]
		membranes	entrapment	glucose sensor	[24,25,26]
		nonwoven fabrics		glucose sensor	[27]
		gels			[28,29,30,31,32,33,34,35,36,37,38]
	*invertase* ^a^	powders			[33]
		membranes from waste silk		glucose sensor	[39,40]
		membranes of regenerated silk fibroin and poly(vinyl alcohol)	entrappment	glucose sensor	[41,42,43]
		films		glucose sensor	[44]
		untreated films		glucose sensor	[9,45]
		composite films of carbon nanotubes and silk fibroin		glucose/O_2_ biofuel cell	[46]
		films on graphene		glucose sensor	[47]
		microneedles		glucose sensor	[48]
2.4	lipase	membranes	entrappment		[49]
		untreated films			[9,45]
		woven fabrics		olive oil hydrolysis and dodecanoic acid esterification	[50]
		gelled silk fibroin-calcium alginate spheres		transesterification of soybean oil with ethanol for biodiesel	[51,52]
		spheres		enzymatic kinetic resolution of halohydrins	[53]
		fibers	glutaraldehyde	sunflower oil hydrolysis for fatty acids	[54]
	*cholesterol oxidase* ^a^	woven mats	N-ethyl-N’-(3-dimethylaminopropyl) carbodimide and N-hydroxysuccinimide ligand chemistry		[55]
2.5	urease	membranes from silk larvae	entrappment	urea electrode	[56]
		membranes		urea removal for wearable artificial kidney	[57]
2.6	uricase	membranes		urate sensor	[58]
2.7	horseradish peroxidase	membranes from silk larvae		H_2_O_2_ sensor	[59]
		membranes from waste silk		H_2_O_2_ sensor	[60,61,62,63,64,65]
	*dehydrogenases* ^a^				[65]
	*glucose oxidase* ^a^				
	*cholesterol oxidase* ^a^				
		membranes of regenerated silk fibroin and poly(vinyl alcohol)		H_2_O_2_ sensor	[66,67,68,69]
	*a bienzyme system of* *horseradish peroxidase* *and* *lactate oxidase* ^a^			lactate sensor	[70]
		scaffolds/sponges	carbodiimide chemistry		[71]
		solutions			[9,45]
		films	entrapment		[72,73]
	*lysozyme* ^a^				
		microspheres	encapsulation		[74]
		inkjet printing			[75]
		optical gratings	entrapment		[76,77]
		Au nanoparticles-silk fibroin		H_2_O_2_ sensor	[78]
		Fe_3_O_4_ nanoparticles-silk fibroin	glutaraldehyde	electroenzymatic oxidation of bisphenol-A	[79]
2.8	catalase	films on graphite		reduction of H_2_O_2_ and NO	[80]
		membranes	adsorption and covalent cross-linking		[81]
		inkjet printing		bubble-propulsive self-motile micro-rockets	[82]
2.9	xanthine oxidase	membranes		electrode for estimating fish freshness	[83]
2.10	tyrosinase	fibrous matrix	glutaraldehyde	large-scale production of L-DOPA	[84]
		composite films of carbon nanotubes-cobalt phthalocyanine-silk fibroin		bisphenol A sensor	[85]
		graphene-silk peptide nanosheets			[86]
2.11	acetylcholinesterase	Au nanoparticles-silk fibroin		pesticide sensor	[87]
		silk fibroin-carbon nanotubes	adsorption		[88]
2.12	neutral protease	nanoparticles	glutaraldehyde	hydrolyze sericin	[89]
	*L-asparaginase* ^a^				
	*β-glucosidase* ^a^				
2.13	α-chymotrypsin	electrospun fibers	glutaraldehyde		[90,91,92]
2.14	amylase	woven fabric	glutaraldehyde	food and pharmaceutical industrial applications	[93]
2.15	organophosphorus hydrolase	gels	entrapment	organophosphate insecticides	[94]
2.16	β-galactosidase	polyacrylonitrile grafted fibers	glyoxal		[95]
2.17	carbonic anhydrase	silk fibroin-coated hydroxyapatite micro-particles	ultrasonically bonded entrapment		[96]
		hydrogels	dual-cross-linking	CO_2_ sequestration	[97]
		hydrogels	Ru(II)-mediated photo-chemical cross-linking		[98]
	*lysozyme* ^a^				
	*xylanase* ^a^				
2.18	laccase	hydrogels	Ru(II)-mediated photo-chemical cross-linking	polymerization of pyrrole	[99]
				grafting of lignin	[100]
2.19	zymolyase	Fe_3_O_4_-embedded silk fibroin microspheres	Ru(II)-mediated photo-chemical cross-linking	disruption of yeast cells	[101]
2.20	L-asparaginase	powders	glutaraldehyde	anti-leukemia	[102,103,104,105]
	*𝛽* *-glucosidase* ^a^				[104]
2.21	phenylalanine ammonia-lyase	microparticles	encapsulation	oral enzyme therapy of phenylketonuria	[106]
2.22	thymidine kinase	recombinant silk-elastin-like protein polymers		viral gene delivery in anticancer treatment	[107,108]

^a^ Similar immobilization methods could be extended to enzymes shown in italic font.

**Table 2:** Silk fibroin-supported non-enzymatic catalysts listed in this review.

Section	Immobilized Non-Enzymatic Catalysts	Explored Applications	References
3.1	core–shell nanostructured gold (Au) colloid–silk fibroin bioconjugate		[109]
	Au nanoparticles/reduced graphene oxide	oxygen reduction reaction (ORR)	[110]
	hybrid wool keratin/Au nanoparticles	sensors for copper ions and dopamine	[111]
	Au nanozyme/bovine serum albumin	H_2_O_2_ sensor	[112]
	millimeter-large Au single crystals		[113]
3.2	palladium (Pd)	chemoselective hydrogenation	[114,115,116]
3.3	iron (Fe)	phenol hydroxylation	[117]
	hematite (α-Fe_2_O_3_)	H_2_O oxidation	[118,119]
	ferriferous oxide (Fe_3_O_4_)	H_2_O_2_ sensor	[120]
3.4	titanium dioxide (TiO_2_) and TiO_2_@Ag nanoparticles	photocatalytic degradation of methylene orange	[121]
	TiO_2_ and Ni-P metallization layer		[122]
3.5	platinum (Pt) nanoparticles	conversion of 4-nitrophenol into 4-aminophenol	[123]
	Pt microspheres on multi-walled carbon nanotubes	H_2_O_2_ sensor	[124]
3.6	zinc oxide (ZnO)	photocatalytic degradation of rhodamine B	[125]
	Au nanoparticles and ZnO nanotubes	H_2_O_2_ sensor	[126]
	ZnO/Au layered structure	solar energy harvesting	[127]
3.7	cupric oxide (CuO)	photocatalytic degradation of Congo Red	[128]
	cuprous oxide (Cu_2_O) embedded in carbon spheres	glucose sensor	[129]
3.8	trimanganese tetraoxide (Mn_3_O_4_)		[130]
	manganese dioxide (MnO_2_)	H_2_O_2_ sensor	[131,132,133,134,135]
